# Homeostatic DNMT3a Activity Is Required to Restore Cognition and Hippocampal DNA Methylation in the 5xFAD Model of Alzheimer’s Disease

**DOI:** 10.14336/AD.2025.0283

**Published:** 2026-06-20

**Authors:** Yanzhi Liu, Chi Him Poon, Long Sum Rachel Tse, Jaydeep Roy, Charlotte Theodora So, Benjamin Chun-Kit Tong, Kwok-Ming Yao, George Lim Tipoe, Harry Steinbusch, Luca Aquili, Allan Kalueff, King-Ho Cheung, Man-Lung Fung, Lee Wei Lim

**Affiliations:** ^1^School of Biomedical Sciences, Li Ka Shing Faculty of Medicine, The University of Hong Kong, Hong Kong SAR, China.; ^2^School of Chinese Medicine, Hong Kong Baptist University, Hong Kong SAR, China.; ^3^Department of Cellular and Translational Neuroscience, School for Mental Health and Neuroscience, Maastricht University, Maastricht, the Netherlands.; ^4^School of Management, Ritsumeikan Asia Pacific University, Beppu, Oita, Japan.; ^5^DrLim Neuromodulation Lab, Department of Biosciences and Bioinformatics, School of Science, Xi’an JiaotongLiverpool University, Suzhou, China.; ^6^Department of Biosciences and Bioinformatics, Suzhou Key Laboratory of Neurobiology and Cell Signaling, School of Science, Xi'an Jiaotong-Liverpool University, Suzhou, China.; ^7^Department of Neurosurgery, Taihe Hospital, Hubei University of Medicine, Shiyan, Hubei, China.

**Keywords:** Alzheimer's disease, Memory, DNA Methylation, L-methionine, Serotonin

## Abstract

Dysregulation of DNA methylation has been implicated in Alzheimer’s disease (AD), making the manipulation of DNA methylation processes a promising therapeutic strategy. DNA methyltransferase 3a (DNMT3a), one of the two *de novo* DNMTs, is involved in learning and memory. However, it remains elusive whether and how alterations in DNMT3a expression contributes to AD pathogenesis. In this study, we investigated the consequences of DNA methylation dysregulations in the hippocampus of 5xFAD mouse model of AD and explored the use of L-methionine (MET) supplement to restore DNA methylation dysregulations. The 5xFAD model exhibited spatial memory impairments accompanied by global DNA hypomethylation and dysregulated hippocampal expression of DNA methyltransferases (DNMT) and demethylases. Prolonged treatment with MET rescued memory deficits, reduced amyloid-β load, decreased neuroinflammation, restored the expression of plasticity-regulating genes and proteins, and enhanced serotonergic neurotransmission. DNMT3a knockdown diminished the pro-cognitive effects of MET and independently impaired spatial memory and hippocampal neuroplasticity in both wildtype and 5xFAD mice. Interestingly, DNMT3a overexpression also had detrimental effects on spatial memory and hippocampal neuroplasticity in both genotypes. Our findings demonstrate that methyl supplementation can be a promising therapeutic strategy for AD patients with brain DNA hypomethylation and that maintaining DNMT3a homeostasis is crucial for normal cognitive functions and the pro-cognitive effects of MET.

## INTRODUCTION

Alzheimer’s disease (AD) is a debilitating neuro-degenerative disorder characterized by progressive cognitive decline. As a multifactorial disease, AD involves complex pathogenic mechanisms. The primary pathological hallmarks of AD include the deposition of β-amyloid (Aβ) plaques, the accumulation of tau neurofibrillary tangles, and widespread neuronal loss [1]. These pathological alterations lead to synaptic dysfunction and the disruption of neurochemical processes essential for memory formation and cognitive function [2, 3].

Accumulating evidence suggests that dysregulation of DNA methylation contributes to AD pathogenesis [4]. DNA methylation refers to the covalent transfer of a methyl group from S-adenyl methionine (SAM) to the C5 position of a cytosine residue to form 5-methylcytosine (5-mC), which occurs predominantly on cytosines in CpG dinucleotides [5, 6]. DNA methylation modulates the expression of genes that are essential for cognitive functions [7-9]. Global and gene-specific DNA methylation changes have been widely reported in AD brains [10-19]. In the hippocampus, Chouliaras et al. reported that 5-mC and 5-hydroxymethylcytosine (5-hmC) immunoreactivity decreased by roughly 20% in AD patients compared to age-matched controls [12]. Similarly, Mastroeni et al. reported that the entorhinal cortex shows more than 2-fold reduction in 5-mC-positive neurons in AD, along with diminished 5-mC staining in microglia and astrocytes [11]. Using a high-throughput array method, Humphries et al. found that genomic CpGs in the temporal pole showed overall hypomethylation in late-onset AD patients compared to controls [13]. Phipps et al. found that nuclear 5-mC and 5-hmC levels are unchanged in pyramidal neurons and interneurons in the inferior temporal gyrus of late-stage AD patients, though extranuclear 5-mC decreases in pyramidal neurons [14]. Astrocytes in this region show reduced nuclear 5-mC and 5-hmC, correlating with amyloid plaque and neurofibrillary tangle burden [14]. Conversely, using the dot-blot method, Bradley-Whitman et al. found that 5-mC and 5-hmC levels in the hippocampus and parahippocampal gyrus were increased in both late-stage AD patients and cognitively normal people with AD histopathology compared to cognitively normal people without AD histopathology [15]. Lashley et al. observed no discernable difference in 5-mC and 5-hmC immunoreactivity in the entorhinal cortex between AD patients and controls [16]. In summary, while many studies reported decreased global DNA methylation in AD brains, others documented either increased or unchanged levels. These apparent discrepancies can be explained by locus-specific methylation changes across the genome, where distinct CpG sites may become either hypermethylated or hypomethylated [17-19]. Such locus specific variations could produce divergent global methylation patterns depending on the brain regions and cell types analyzed. Importantly, despite reported variabilities, the collective evidence strongly implicates DNA methylation dysregulation as a consistent feature of AD neuropathology.

DNA methyltransferases (DNMTs) regulate DNA methylation, with DNMT1 maintaining methylation patterns during replication and in postmitotic cells [20], while DNMT3a and DNMT3b catalyze *de novo* methylation, exhibiting distinct genomic targeting preferences [21]. Active DNA demethylation is mediated by the Ten-eleven translocation (TET) enzymes, which oxidize 5-mC to initiate demethylation [22]. Methyl-binding domain (MBD) proteins interpret DNA methylation signals to modulate gene transcription [23]. The dynamic interplay between DNMTs, TETs, and MBDs in regulating methylation-demethylation cycles and gene expression remains an active area of investigation. As discussed above, dysregulated DNA methylation is implicated in the pathogenesis of AD. A DNMT3a single nucleotide polymorphism (SNP) that potentially leads to an alternative splice variant was shown to be associated with cognitive decline in mild cognitive impairment patients [24]. Two transcription regulating SNPs in the DNMT3b gene were found to be associated with AD [25]. Similarly, TET1 loss-of-function genetic variants are associated with early-onset AD [26], and heterozygous knockout of TET1 in 5xFAD mice worsened fear-conditioning memory and Aβ deposition [26]. These findings collectively demonstrate that dysregulations in DNA methylation writers (DNMTs) and erasers (TETs) contribute to AD pathogenesis.

DNA methylation is highly dependent on the availability of SAM, which donates methyl groups in methylation reactions. In humans, SAM is synthesized as part of the intricate nutrient metabolic network known as one-carbon metabolism [27]. Studies have found that perturbations in the homeostasis of this network contribute to the pathogenesis of AD. In the elderly, one established risk factor for AD is elevated plasma homocysteine (HCY) level, as increased HCY level suppresses methylation reactions [28, 29]. Moreover, deficiencies in folate, vitamin B12, and B6 are also recognized as risk factors for AD [30-34], as they lead to elevated HCY level. Patients with AD have also been shown to have decreased SAM levels [35, 36]. Subsequently, studies have investigated the use of nutrient supplementations to maintain normal DNA methylation profiles as a treatment for AD. Two early clinical trials showed that dietary supplementation of SAM had pro-cognitive effects in AD patients [37, 38]. Despite accumulating evidence supporting the therapeutic potential of maintaining DNA methylation homeostasis in AD, there is still limited understanding of the cellular and molecular effects of DNA methylation dysregulations in the brain. How DNA methylation dysregulations affect memory and emotion regulating processes such as synaptic plasticity and serotonergic (5-HT) neurotransmission in AD remains elusive.

Here, we investigated the role of DNA methylation dysregulations in the pathogenesis of AD using the 5xFAD mouse model. We found that 5xFAD mice exhibited early onset cognitive impairments accompanied by global DNA hypomethylation and dysregulated hippocampal expression of DNA methyltransferases (DNMT) and demethylases. The global DNA hypomethylation phenotype observed in 5xFAD mice mirrors findings from a substantial subset of AD patients [11-13]. This correspondence underscores the translational value of investigating both the molecular mechanisms driving brain DNA hypomethylation in this model and potential therapeutic strategies to reverse these maladaptive epigenetic changes. Such research holds significant promise for developing targeted interventions applicable to human AD pathology. Here, we found that prolonged treatment with L-methionine (MET), a precursor of SAM, rescued hippocampal-dependent memory deficits, ameliorated AD neuropathology, enhanced the expression of plasticity-regulating genes via a DNA methylation-dependent mechanism, restored protein expression and phosphorylation in cAMP response element binding protein (CREB)-brain-derived neurotrophic factor (BDNF) and calcineurin (CaN)-glycogen synthase kinase-3β (GSK3β) signaling pathways, and facilitated 5-HT neurotransmission. Our study revealed that normal hippocampal expression of DNMT3a, one of two *de novo* DNMTs, is necessary for proper hippocampal function, as DNMT3a knockdown (KD) impaired memory and attenuated the beneficial effects of MET. Moreover, hippocampal DNMT3a overexpression (OE) impaired memory in both wildtype (WT) and 5xFAD mice. Our findings suggest that maintaining physiological homeostasis of DNA methylation/demethylation processes is crucial for normal memory function. These results suggest that methyl supplementation may represent a promising therapeutic strategy for AD patients exhibiting brain DNA hypomethylation.

## MATERIALS AND METHODS

### Animals

Male 5xFAD mice (4.5 months old) carrying the human APP transgene with Swedish, Florida and London mutations and PSEN1 transgene with M146L and L286V mutations were obtained from The Jackson Laboratory. The animals were maintained in a controlled environment at 25°C under a 12-h/12-h light/dark cycle (lights on at 10:00) with food and water available *ad libitum*. All experimental procedures were approved by the Committee on the Use of Live Animals in Teaching and Research (CULATR) of The University of Hong Kong (Ref: 4549-17). All experimental procedures followed recommendations in the ARRIVE guidelines.

### Drug administration

Transgenic (5xFAD) and non-transgenic wildtype (WT) mice received subcutaneous injections of 200 mg/kg L-methionine (Sigma-Aldrich) dissolved in saline (MET groups) or saline (SAL groups) twice daily (09:00/15:00) for 1.5 months before the start of the behavioral tests, and injections were continued throughout the behavioral testing. The MET dosage has been shown to affect global DNA methylation status [39]. On the last day of treatment, animals were injected with MET/SAL 2 h before being sacrificed.

### Behavioral testing

#### Y maze forced alternation

The Y maze test was performed as previously described [40] with minor modifications. The Y maze apparatus consisted of three arms (35 x 6 x 15 cm) made of white plastic joined at the middle to form a ‘Y’ shape. The interior of the arms was identical and did not include any intramaze spatial cues that would influence the rodents’ curiosity to explore novel areas. The Y maze was divided into a start arm, a familiar arm, and a novel arm. In the acquisition phase, the novel arm was closed, and the mouse was placed in the start arm and allowed to freely explore the maze for 5 min. In the retrieval phase following a 30-min retention period, the novel arm was opened, and the animal was returned to the start arm and allowed to explore the entire maze for 5 min. The experiment was recorded using an overhead camera connected to the ANY-maze system (Stoelting, USA) to track the animal’s movements throughout the test.

#### Y maze spontaneous alternation

The Y maze spontaneous alternation test was performed as previously described [41]. Mice were individually placed in the center of the maze with all three arms open and allowed to freely explore the maze for 5 min. The percentage of spontaneous alternations exhibited by the animal was measured as the number of alternations made (entries into three different arms consecutively) divided by the total possible alternations made (the total number of arm entries minus 2) and multiplied by 100.

#### Open field test (OFT)

The OFT test was used to investigate anxiety and locomotor activity and was performed as previously described [42]. The open squared arena consisted of a Plexiglass box (40 x 40 x 40 cm) with an open top and white floor. Mice were individually placed in the open arena and allowed to freely explore for 5 min. The experiment was recorded using an overhead camera connected to the ANY-maze system (Stoelting) to track the animal’s movements throughout the test.

#### Morris water maze (MWM)

The MWM was performed as previously described [43] with minor modifications. Briefly, the test was performed in a black circular pool (150 cm diameter) filled with water maintained at a temperature of 25±1°C. Mice were tested for their ability to locate a submerged circular, transparent escape platform (2 cm below water surface) placed 35 cm from the edge of the pool. In the acquisition phase, mice were trained to locate the submerged platform from randomized starting positions equidistant from the platform in four trials per day for 4 consecutive days. Each animal was allowed 1 min to search for the submerged platform. If the mouse failed to locate the submerged platform within 1 min, it was gently guided to the platform and allowed to stay on the platform for 15 s. In the probe phase at 24 h after the last training session, the platform was removed, and each mouse was allowed 1 min to explore the pool. The time to locate the imaginary platform was recorded.

### rAAV

To knock down Dnmt3a, a vector was constructed containing a U6 promoter to drive shRNA expression and a human cytomegalovirus (CMV) promoter to drive eGFP expression. The Dnmt3a-specific shRNA sequence was “acgggcagctatttacagagc”, which has been previously experimentally verified [44]. The non-targeting scrambled control shRNA sequence was “cctaag gttaagtcgccctcg”. To overexpress Dnmt3a, we used a recombinant adeno-associated viral (rAAV) vector containing the human synapsin 1 (hSyn) promoter to drive neuronal-specific expression of 3xFLAG epitope-tagged mouse Dnmt3a. A vector containing mCherry driven by the hSyn promoter was used as the control. Schematics of the rAAV vectors are shown in Supplementary Fig. 3A and 4A. The serotype of the rAAV was 2/9. Viral vector construction and packaging were carried out by Obio Technology, Shanghai, China. Viral titers were 1.06 x 10^13^ viral particles mL^-1^ for rAAV-Dnmt3a shRNA, 5.63 x 10^12^ viral particles mL^-1^ for rAAV-shRNA Ctrl, 1.98 x 10^13^ viral particles mL^-1^ for rAAV-3xFLAG-Dnmt3a, and 1.57 x 10^13^ viral particles mL^-1^ for rAAV-mCherry,

### Stereotaxic surgery

Mice received bilateral infusion of rAAV into the CA1-2 region of the dorsal hippocampus (stereotaxic coordinates: A/P: - 2.0 mm, M/L: 1.5 mm, and D/V: 1.5 mm relative to the Bregma). Briefly, animals were anesthetized by isoflurane inhalation and 1.5 μL of virus was infused into each hemisphere. The virus was delivered via a 10-μL Hamilton syringe at a steady speed of 0.4 μL/min using a syringe pump (Harvard Apparatus). The infusion needle was left in place for 10 minutes after injection to ensure fluid diffusion. For mice receiving rAAV-shRNA Ctrl or rAAV-Dnmt3a shRNA infusion, MET or SAL daily injections commenced 14 days after surgery to allow sufficient time for gene knockdown. Mice receiving rAAV-mCherry or rAAV-3xFLAG-Dnmt3a infusion were handled daily by experimenters.

### Electrophysiology

#### Acute hippocampal slice preparation

Mice were sacrificed by cervical dislocation. The brains were quickly collected and submerged in ice-cold sucrose supplemented with cutting solution (in mM: NaCl 64, KCl 2.5, KH_2_PO_4_ 1.25, CaCl_2_ 0.5, MgSO_4_ 10, NaHCO_3_ 26, D-glucose 10, sucrose 120, saturated with 95% O_2_ and 5% CO_2_, pH 7.3). The cerebellum and brain stem were removed by blocking using a razor blade. The rest of the brain was then glued onto a vibratome (5100 mz, Campden Instrument) stage and submerged in ice-cold modified sucrose supplemented with Krebs Ringer solution bubbled with 95% O_2_ and 5% CO_2_. The left and right hemispheres were separated. Serial coronal sections (350 μm) were cut and sections containing the dorsal hippocampus were collected and incubated in recording solution (in mM: NaCl 120, KCl 3.5, KH_2_PO_4_ 1.25, CaCl_2_ 2.5, MgSO_4_ 1.5, NaHCO_3_ 26, D-glucose 10, saturated with 95% O_2_ and 5% CO_2_, pH 7.3) at 32°C for at least 1 h for equilibration. Sections were transferred into recording chambers lined with multi-electrode arrays (MED-PG501A, Alpha MED Scientific Inc.) and continuously perfused with recording solution at room temperature.

#### Recording

Schaffer collaterals of CA3 were stimulated and field excitatory postsynaptic potentials (fEPSPs) were recorded in the stratum radiatum of CA1-2 using the MED64-Quad II system (Alpha MED Scientific Inc.). Placement of the stimulating and recording electrodes is shown in Fig. 9A. Input-output (I/O) curves were first obtained by applying stimuli at increasing intensity (10 - 80 μA). The stimulus intensity that elicited a half maximum response was used to invoke fEPSPs in the following experiments. Paired-pulse facilitation (PPF) was measured by applying paired stimuli with interstimulus intervals of 10, 20, 50, 100, 150, and 200 ms. Paired-pulse ratios were calculated as the slope of the second fEPSP divided by the slope of the first fEPSP. Long-term potentiation (LTP) was induced following a 20-min stable baseline using 10 bouts of theta-burst stimulations (TBS) delivered over 2 s. Each bout consisted of five pulses at 100 Hz and was separated from the previous/next bout by 150 ms. fEPSPs were recorded for 60 min after TBS. The magnitude of LTP was quantified by dividing the slope of post-TBS fEPSPs by the averaged slope during baseline recording. Data were analyzed using the Mobius software (Alpha MED Scientific Inc.).

### Immunohistochemistry and immunofluorescence

On the last day of treatment, mice received MET/SAL injections 2 h before being sacrificed (pentobarbital sodium, i.p.). Mice were immediately decapitated and brains were extracted, snap-frozen in liquid nitrogen, and stored at -80°C until further processing for molecular analysis. Alternatively, mice were perfused transcardially with ice-cold Tyrode solution followed by 4% paraformaldehyde in PBS. Brains were then removed and fixed in 4% paraformaldehyde in PBS overnight before being transferred to 15% and 30% sucrose in PBS sequentially and kept in the solution until they sank to the bottom. Brain tissues were then snap-frozen in liquid nitrogen and coronal sections (20 μm) were cut using a CryoStar NX50 Cryostat (Thermo Scientific, USA). At least three hippocampal sections per animal were imaged and analyzed for each set of markers.

#### Immunohistochemistry

**Figure 1.**
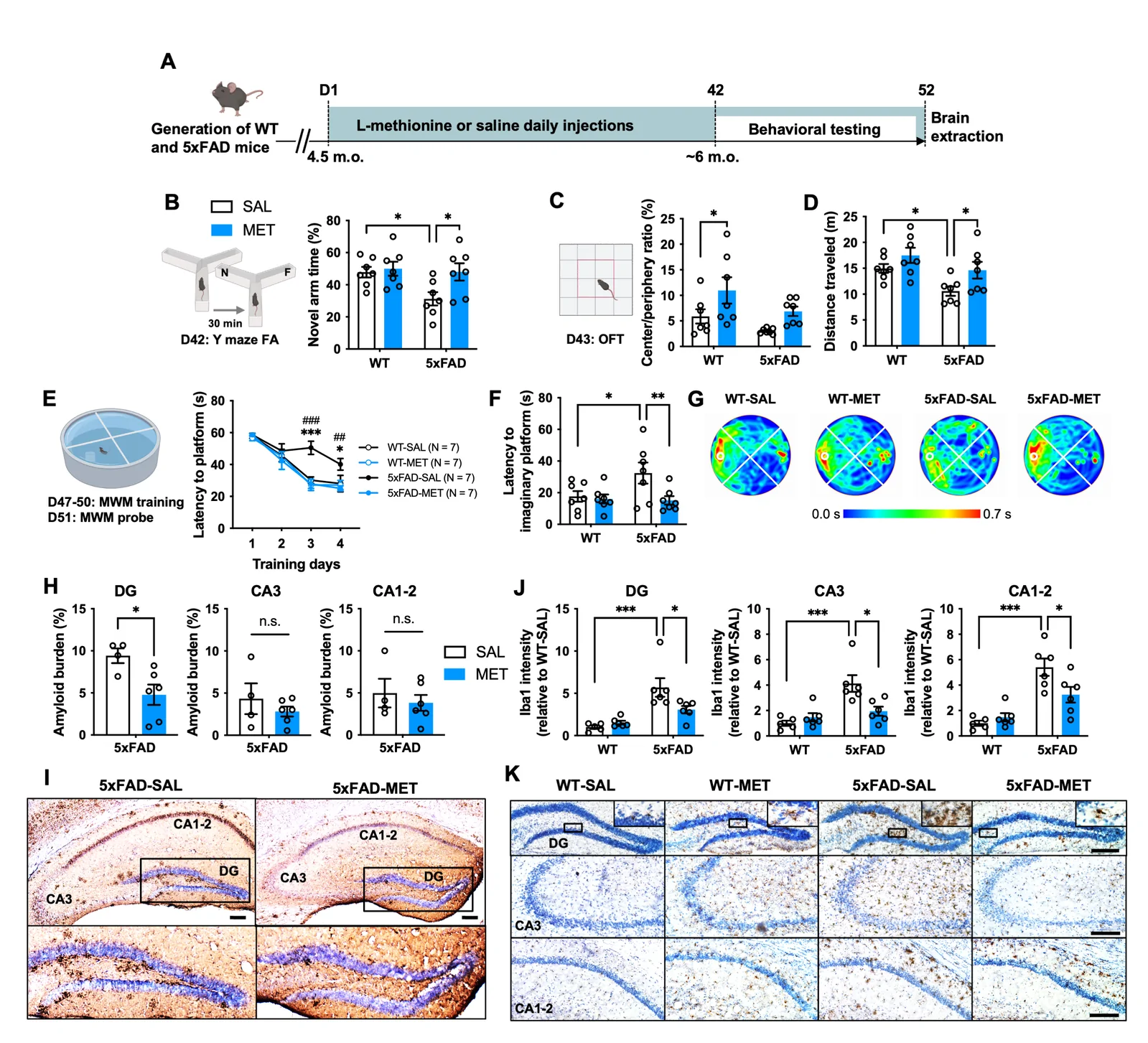
**Protracted MET treatment enhances cognitive performance in 5xFAD mouse model**. (**A**) Experimental timeline. WT and 5xFAD mice of 4.5 months old (m.o.) were used. Mice were roughly 6 m.o. at the start of behavioral tests. (**B**) Percentage of time spent in the novel arm in the Y maze forced alternation (FA) test (N = 7 per group) (two-way ANOVA: genotype: *F*_1,24_ = 4.56, *p* = 0.043; treatment: *F*_1,24_ = 4.70, *p* = 0.040; Fisher’s LSD post-hoc: WT-SAL vs 5xFAD-SAL: *p* = 0.013, 5xFAD-SAL vs 5xFAD-MET: *p* = 0.012). (**C-D**) Open field test (N = 7 per group). (**C**) Center-to-periphery ratio (two-way ANOVA: treatment: *F*_1,24_ = 8.425, *p* = 0.008; Fisher’s LSD post-hoc: WT-SAL vs WT-MET: *p* = 0.029). (**D**) Total distance travelled (two-way ANOVA: genotype: *F*_1,24_ = 8.39, *p* = 0.008; treatment: *F*_1,24_ = 6.88, *p* = 0.015; Fisher’s LSD post-hoc: WT-SAL vs 5xFAD-SAL: *p* = 0.021, 5xFAD-SAL vs 5xFAD-MET: *p* = 0.032). (**E-F**) Morris Water Maze (MWM) (N = 7 per group). (**E**) Latency to reach the platform location during training days [three-way ANOVA: genotype × treatment: *F*_1,48_ = 7.67, *p* = 0.008; Fisher’s LSD post-hoc: WT-SAL vs 5xFAD-SAL (indicated by *): *p* < 0.001 on day 3 and day 4, 5xFAD-SAL vs 5xFAD-MET (indicated by #): *p* < 0.001 on day 3 and day 4]. (**F**) Latency to cross imaginary platform location during probe test (two-way ANOVA: treatment: *F*_1,24_ = 4.93, *p* = 0.036; genotype × treatment: *F*_1,24_ = 3.42, *p* = 0.077; Fisher’s LSD post-hoc: WT-SAL vs 5xFAD-SAL: *p* = 0.021, 5xFAD-SAL vs 5xFAD-MET: *p* = 0.008). (**G**) Cumulative swimming tracks by each group during MWM probe test. (**H**) Amyloid-β (Aβ) burden in the DG, CA3 and CA1-2 subregions of dorsal hippocampus of 5xFAD mice treated with SAL (N = 4) or MET (N = 6) (Man-Whitney U tests: DG: *U* = 2, *p* = 0.038). (**I**) Representative images of Aβ staining in the hippocampus of 5xFAD mice (scale bar: 200 μm). (**J**) Expression of microglial marker Iba1 in the DG, CA3 and CA1-2 subregions of dorsal hippocampus of WT and 5xFAD mice treated with SAL or MET (N = 5-6 per group) (two-way ANOVA: DG: genotype: *F*_1,19_ = 23.52, *p* < 0.001; genotype × treatment: *F*_1,19_ = 6.05, *p* = 0.024; CA3: genotype: *F*_1,19_ = 17.08, *p* < 0.001; genotype × treatment: *F*_1,19_ = 9.15, *p* < 0.001; CA1-2: genotype: *F*_1,19_ = 35.43, *p* < 0.001; genotype × treatment: *F*_1,19_ = 6.41, *p* = 0.020; Šídák’s post-hoc: WT-SAL vs 5xFAD-SAL: *p*-values < 0.001; 5xFAD-SAL vs 5xFAD-MET: DG: *p* = 0.047, CA3: *p* = 0.011, CA1-2: *p* = 0.042). (**K**) Representative images of Iba1 staining in the hippocampus (scale bars: 200 μm for DG and 100 μm for CA3 and CA1-2). Data are presented as means ± s.e.m. *p < 0.05, ** *p* < 0.01, *** *p* < 0.001, n.s., not significant.

Free-floating tissue sections containing the dorsal hippocampus (approximately Bregma -1.58 to -2.30 mm) were blocked with 0.5% H_2_O_2_ solution and 1% BSA, and then incubated with the primary antibodies including mouse anti-Aβ, Clone 4G8 (1:500, 800703, Biolegend), goat anti-Iba1 (1:1000, ab5076, Abcam) overnight at 4°C. After washing, sections were incubated with corresponding biotinylated goat anti-mouse IgG (1:1000; BA9200, Vector Laboratories) or horse anti-goat IgG (1:1000; BA9500, Vector Laboratories) for 1.5 h at room temperature. The signal was amplified using Vectastain Elite ABC-HRP Kit (PK-6100, Vector Laboratories) and finally visualized by 3, 3'-diaminobenzidine (DAB) supplemented with nickel chloride. Sections were mounted on SuperFrost microscope slides and counterstained with hematoxylin (Sigma-Aldrich), followed by dehydration with alcohol and xylene, and then cover-slipped with Permount solution (Thermo Fisher Scientific). Sections were imaged under brightfield illumination using an Olympus BX51 microscope. Aβ and Iba1 immunoreactivities were quantified as the percentage of the area of interest covered by immune-positive signals. The Iba1 expression values of all experimental group values were calculated as fold changes relative to their respective control group means. Specifically, we used: WT-SAL group as control for Figures 1; WT-SAL-shRNA Ctrl group for Figures 5; WT-AAV Ctrl group for Figures 7. The specificity of the primary antibodies was confirmed by the absence of detectable signal in secondary antibody-only controls.

#### Immunofluorescence

Free-floating tissue sections were incubated in sodium borohydride (0.1%, w/v) in 0.1 M PBS for 30 min to reduce autofluorescence, followed by pretreatment for antigen retrieval (5mC: 10 mM citrate buffer, pH 3.0, steamed in a gently rotating water bath at 100°C for 20 mins; 5-HT: 10 mM citrate buffer, pH 6.0, steamed in a gently rotating water bath at 80°C for 20 min). Sections were then blocked with 5% BSA for 30 min before incubating with primary antibodies including mouse anti-5mC (1:500, C15200081, Diagenode), and rabbit anti-5-HT (1:2000, gifted by Prof. H.W.M. Steinbusch). Primary antibodies were visualized with secondary antibodies including horse anti-mouse IgG H+L DyLight 488 (1:500, DI2488, Vector Laboratory), goat anti-rabbit IgG H&L DyLight 594 (1:500, DI1594, Vector Laboratory), and donkey anti-rabbit 647 (1:500, A31573, Thermo Fisher Scientific). DAPI counterstaining was achieved by embedding sections in mounting medium containing DAPI (Fluoromount-G, Invitrogen). Images were acquired under a ZEISS LSM880 confocal microscope with a 20x air lens using standard confocal mode. Each region was captured as 15-20 Z-stacks with an interstack interval of 0.8 μm. The laser intensity of each channel was kept constant for all samples. The specificity of the primary antibodies was confirmed by the absence of detectable signal in secondary antibody-only controls.

### Global DNA methylation quantification

Measurement of global DNA methylation level was performed as previously described [45]. Briefly, genomic DNA was extracted from hippocampal tissue using the QIAamp Fast DNA Tissue Kit (Qiagen). DNA methylation was quantified by colorimetric assessment using MethylFlashTM Global DNA methylation ELISA Easy kit (Epigentek). Total DNA methylation percentage was determined using a standard curve generated using methylated and unmethylated DNA standards (Epigentek). DNA methylation percentage for each animal group was calculated after normalizing to the WT-SAL group.

### Reverse-transcription real-time PCR

**Figure 2.**
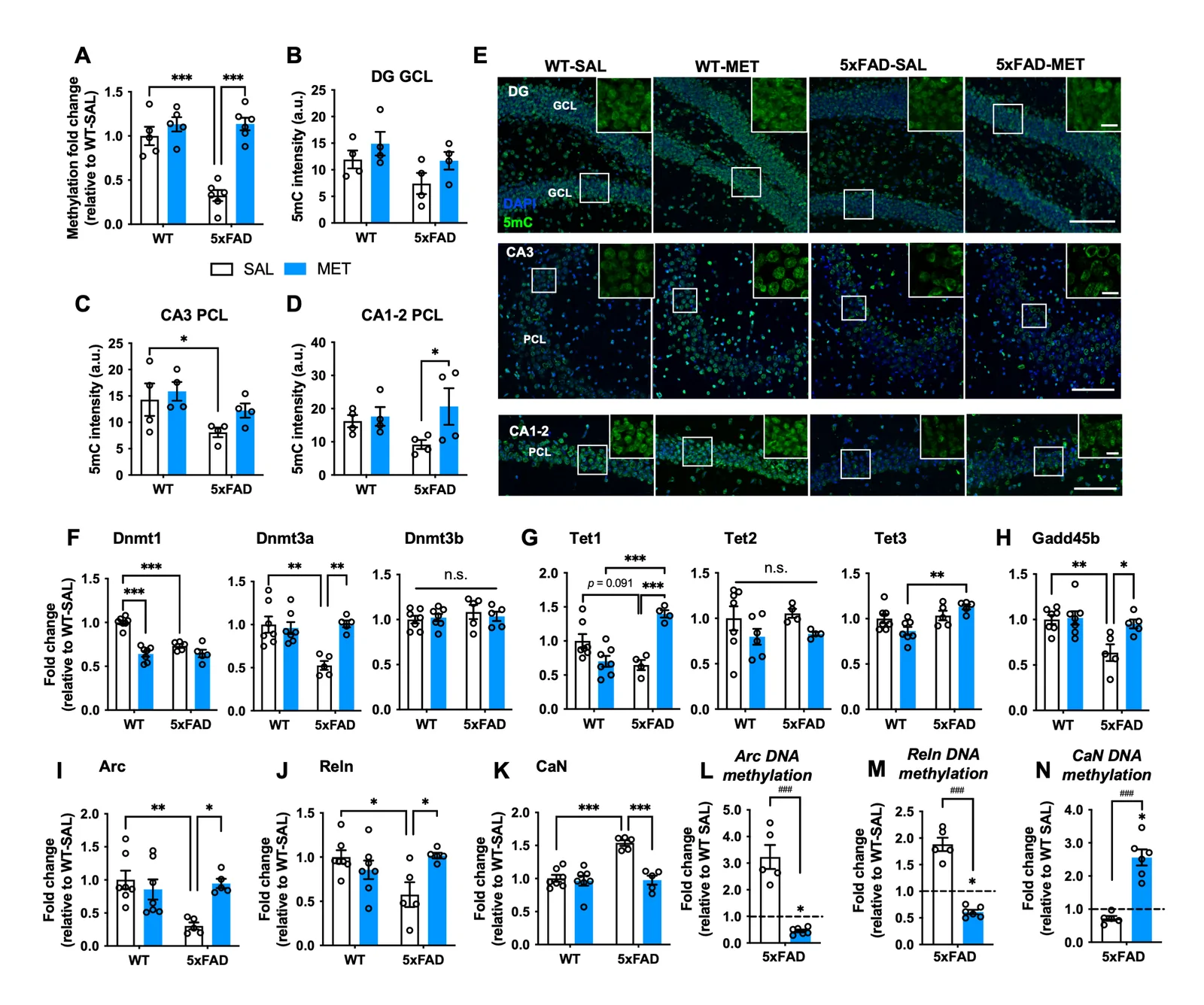
**Protracted MET treatment restored hippocampal global DNA methylation, plasticity, and memory-related gene expressions through promoter DNA methylation modulation in 5xFAD mice**. (**A**) Global DNA methylation levels in the whole dorsal hippocampus of WT (N = 5 per group) and 5xFAD mice (N = 6 per group) treated with SAL or MET measured by ELISA (two-way ANOVA: genotype: *F*_1,18_ = 18.02, *p* < 0.001; treatment: *F*_1,18_ = 35.41, *p* < 0.001; genotype × treatment: *F*_1,18_ = 18.34, *p* < 0.001; Šídák’s post-hoc: WT-SAL vs 5xFAD-SAL: *p* < 0.001, 5xFAD-SAL vs 5xFAD-MET: *p* < 0.001). (**B-D**) 5mC immunofluorescence signals were quantified in the DG granule cell layer (GCL) (B) and CA3/CA1-2 pyramidal cell layer (PCL) (C-D) of the dorsal hippocampus (N = 4 per group) (two-way ANOVA: DG: genotype: *F*_1,12_ = 4.14, *p* = 0.065, treatment: *F*_1,12_ = 3.65, *p* = 0.081; CA3: genotype: *F*_1,12_ = 6.37, *p* = 0.027, Šídák’s post-hoc: WT-SAL vs 5xFAD-SAL: *p* = 0.044; CA1-2: treatment: *F*_1,12_ = 3.82, *p* = 0.074, Šídák’s post-hoc: 5xFAD-SAL vs 5xFAD-MET: *p* = 0.030). (**E**) Representative 5mC immunofluorescence images in the dorsal hippocampal subfields of WT or 5xFAD mice treated with either SAL or MET (scale bars: 100 μm for DG and CA3, 70 μm for CA1-2 and 15 μm for insets). (**F**) mRNA levels of DNA methyltransferases Dnmt1, Dnmt3a and Dnmt3b in SAL or MET treated WT (N = 7 per group) or 5xFAD mice (N = 5 per group) (two-way ANOVA: Dnmt1: genotype: *F*_1,20_ = 17.77, *p* < 0.001, treatment: *F*_1,20_ = 50.37, *p* < 0.001, genotype × treatment: *F*_1,20_ = 19.01, *p* < 0.001, Šídák’s post-hoc: WT-SAL vs 5xFAD-SAL: *p* < 0.001, WT-SAL vs WT-MET: *p* < 0.001; Dnmt3a: genotype: *F*_1,20_ = 8.05, *p* = 0.010, treatment: *F*_1,20_ = 8.67, *p* = 0.008, genotype × treatment: *F*_1,20_ = 8.67, *p* = 0.008, Šídák’s post-hoc: WT-SAL vs 5xFAD-SAL: *p* = 0.001, 5xFAD-SAL vs 5xFAD-MET: *p* = 0.003). (**G-H**) mRNA levels of DNA demethylases Tet1-3 (G) and Gadd45b (H) in SAL or MET treated WT (N = 6-7 per group) or 5xFAD mice (N = 4-5 per group) (two-way ANOVA: Tet1: treatment: *F*_1,18_ = 5.84, *p* = 0.027, genotype × treatment: *F*_1,18_ = 31.49, *p* < 0.001, Šídák’s post-hoc: WT-SAL vs 5xFAD-SAL: *p* = 0.091, 5xFAD-SAL vs 5xFAD-MET: *p* < 0.001; Tet3: genotype: *F*_1,20_ = 10.26, *p* = 0.005, Šídák’s post-hoc: WT-MET vs 5xFAD-MET: *p* = 0.004; Gadd45b: genotype: *F*_1,20_ = 9.96, *p* = 0.005, treatment: *F*_1,20_ = 6.00, *p* = 0.024, genotype × treatment: *F*_1,20_ = 4.83, *p* = 0.040, Šídák’s post-hoc: WT-SAL vs 5xFAD-SAL: *p* = 0.007, 5xFAD-SAL vs 5xFAD-MET: *p* = 0.038). (**I-K**) mRNA levels of Arc (I), Reln (J), and CaN) (K) in SAL or MET treated WT (N = 7 per group) or 5xFAD mice (N = 5 per group) (two-way ANOVA: Arc: genotype: *F*_1,20_ = 5.53, *p* = 0.030, genotype × treatment: *F*_1,20_ = 9.31, *p* = 0.006; Reln: genotype × treatment: *F*_1,20_ = 9.00, *p* = 0.007; CaN: genotype: *F*_1,20_ = 18.16, *p* < 0.001, treatment: *F*_1,20_ = 21.71, *p* < 0.001, genotype × treatment: *F*_1,20_ = 16.82, *p* < 0.001; Šídák’s post-hoc: WT-SAL vs 5xFAD-SAL: Arc: *p* = 0.006, Reln: *p* = 0.036, CaN: *p* < 0.001; 5xFAD-SAL vs 5xFAD-MET: Arc: *p* = 0.023, Reln: *p* = 0.046, CaN: *p* < 0.001). (**L-N**) Effects MET on promoter DNA methylation levels of Arc, Reln and CaN in the hippocampus of 5xFAD mice (* indicates significant differences comparing with WT-SAL) (N = 5-7 per group) (Kruskal-Wallis H tests: Arc: *H*(2) = 14.24, *p* < 0.001, Reln: *H*(2) = 14.24, *p* < 0.001, CaN: *H*(2) = 13.07, *p* < 0.001; Dunn’s multiple comparison tests: WT-SAL vs 5xFAD-SAL: Arc: *p* = 0.072, Reln: *p* = 0.072; WT-SAL vs 5xFAD-MET: Arc: *p* = 0.040, Reln: *p* = 0.040, CaN: *p* = 0.026; 5xFAD-SAL vs 5xFAD-MET: *p*-values < 0.001). Data are presented as means ± s.e.m. *p < 0.05, ** *p* < 0.01, ***p < 0.001. n.s., not significant.

Total RNA was isolated from hippocampal tissue using Allprep-DNA/RNA/miRNA Universal Kit (Qiagen) according to the manufacturer’s instructions. The concentration of the obtained RNA was measured by Nanodrop. RNA was reverse transcribed to cDNA using PrimeScript RT reagent kit with gDNA eraser (Takara Bio, USA). Real-time qPCR was performed using a LightCycler 480II (Roche) for DNA methyltransferases (Dnmt1, Dnmt3a, Dnmt3b) and DNA demethylases (Growth arrest and DNA-damage inducible 45b, Gadd45b; ten-eleven translocation enzymes, Tet1-3), methylated DNA binding protein (methyl CpG binding protein 2, MeCP2), neuroplasticity-associated genes (Calcineurin, CaN; activity-regulated cytoskeleton-associated protein, Arc; Reelin, Reln), Gα_s_/Gα_q_-coupled 5-HT receptors (Htr2a, Htr2b, Htr2c, Htr4, Htr6, Htr7), Gα_i/o_-coupled 5-HT receptors (Htr1a, Htr1b, Htr1d, Htr1f, Htr5a, Htr5b) and Htr3a. The primer sequences are listed in Supplementary Table 1. Samples were analyzed in triplicate and loading was confirmed by the amplification of Gapdh as the house-keeping gene. The Ct value was selected from the linear phase of the fluorescence curve. Samples were normalized to Gapdh and the gene expressions of all experimental groups were calculated as fold changes relative to their respective control group means using the 2^-DDCT^ method. Specifically, we used: WT-SAL group as control for qPCR results in Figures 2 and 4; WT-SAL-shRNA Ctrl group for Figures 5 and 6; WT-AAV Ctrl group for Figures 7 and 8.

### Methylation-specific real-time PCR

Genes that showed significant changes in the RT-qPCR analysis were subsequently subjected to methylation-specific real-time PCR (MSP). Genomic DNA was first extracted from hippocampal tissue using Allprep-DNA/RNA/miRNA Universal Kit (Qiagen) according to the manufacturer’s instructions. The purified DNA was then subject to bisulfite modification (Methylamp DNA modification kit, Epigentek). Quantitative real-time PCR was performed to determine the DNA methylation status of candidate genes using methylation-specific PCR primers designed using the Methprimer software [46]. The primer sequences are listed in Supplementary Table S2. DNA amplification was performed using Methylamp MS-PCR fast kit (Epigentek). Reactions were performed in a LightCycler 480II (Roche). The thermal profile was 95°C for 7 min for DNA denaturation and enzyme activation, and 45 cycles of 95°C for 10 sec, 55°C for 10 sec, and 72°C for 8 sec for DNA amplification, followed by fluorescence detection. Samples were analyzed in triplicate and loading was confirmed by the amplification of Gapdh as the house-keeping gene. The Ct value was selected from the linear phase of the fluorescence curve. Percentage methylation in each sample was calculated by the following equation:

%Methylation=100/[1+2^DCt(meth-unmeth)^]%.

### Protein preparation and Western blot analysis

The dorsal hippocampus was quickly gross-dissected on ice and homogenized in cold lysis buffer (0.15 M NaCl, 0.05 M Tris-HCl, 1% Triton x100, 0.1% sodium dodecyl sulfate, 0.5% sodium deoxycholate, 1 mM EDTA) containing Halt protease and phosphatase inhibitor cocktail (Thermo Scientific). Homogenates were incubated at 4^o^C for 30 min, centrifuged at 12000 g for 20 min, and supernatants were collected and stored at -80°C until use. Protein concentration of each sample was measured by Bradford assay (Bio-rad). Total protein (10 µg) from each sample were separated on 10% SDS-PAGE gels and transferred to PVDF membranes. The membranes were blocked with 5% non-fat milk or 5% BSA in TBS at room temperature for 1 h and then incubated with primary antibodies overnight at 4^o^C. Antibodies used included BDNF (1:1000, NB100-98682, Novus Biologicals), PKA C-α (1:1000, 4782, Cell Signaling Technology), pPKA C^Thr197^ (1:1000, 4781, Cell Signaling Technology), AKT (1:1000, 4691, Cell Signaling Technology), pAKT^S473^ (1:1000, 9271, Cell Signaling Technology), ERK1/2 (1:1000, 9102, Cell Signaling Technology), pERK1/2 (1:1000, 9101, Cell Signaling Technology), synaptophysin (1:1000, 5461, Cell Signaling Technology), PSD-95 (1:1000, ab18258. Abcam), MeCP2 (1:1000, NB600-1101, Novus Biologicals), pMeCP2^S421^ (1:1000, NBP2-29524, Novus Biologicals), DNMT3a (1:1000, 3598, Cell Signaling Technology), Calcineurin A (1:1000, 2614, Cell Signaling Technology), GSK3β (1:1000, 9315, Cell Signaling Technology), pGSK3β^S9^ (1:1000, 9336, Cell Signaling Technology), CREB (1:1000, 9104, Cell Signaling Technology), pCREB^S133^ (1:1000, MA511192, Life Technologies), and GAPDH (1:1000, 2118, Cell Signaling Technology). The membrane was washed and subsequently probed with HRP-conjugated goat anti-rabbit IgG (1:2000, 31460, Thermo Fisher Scientific) for 1 h. The signal was visualized using Clarity Western ECL Substrate (Bio-rad) and imaged using the ChemiDoc Imaging system (Bio-rad). Samples were normalized to GAPDH and the protein expressions of all experimental groups were calculated as fold changes relative to the WT-SAL group. The specificity of the primary antibodies was confirmed by the absence of detectable signal in secondary antibody-only controls.

### Data analysis

IBM SPSS Statistics 25 was used for the statistical analyses. All data were presented as mean ± s.e.m. Outliers, if any, were removed upon inspection of boxplots. Normality was assessed and confirmed by the Shapiro-Wilk test. Homogeneity of variance was confirmed via Levene's test. MWM performance on training days of different groups was analyzed by three-way analysis of variance (ANOVA) (genotype × treatment × training day) followed by simple effects analysis. Results of other behavioral tests were analyzed by two-way ANOVA (genotype × treatment, genotype × rAAV, or rAAV × treatment) followed by Šídák’s or Fisher’s LSD post-hoc multiple comparison tests. Molecular assay data were analyzed by two-way ANOVA (genotype × treatment, genotype × rAAV, or rAAV × treatment, followed by Šídák’s or Fisher’s LSD post-hoc tests). 4G8 staining results were analyzed using either the Mann-Whitney U test (two-group comparisons) or two-way ANOVA with Šídák’s post-hoc tests (four-group comparisons). Promoter DNA methylation levels were analyzed using either Kruskal-Wallis H test with Dunn’s multiple comparison tests (three-group comparisons) or two-way ANOVA with Šídák’s post-hoc tests (four-group comparisons). Changes in fEPSP slopes before and after TBS in each group were assessed via two-tailed paired t-tests. Details about statistical tests, post-hoc comparisons and post-hoc adjustments for each figure are listed in Supplementary Table 3. Significance was defined as p < 0.05.

## RESULTS

### Prolonged MET treatment normalizes the behavioral alterations in 5xFAD mice

To investigate if MET can restore hippocampal-dependent memory deficits in 5xFAD mice, we administered MET or saline (SAL) in 4.5-month-old 5xFAD and WT mice for 1.5 months before behavioral testing (Fig. 1A). We observed that SAL-treated 5xFAD mice (5xFAD-SAL) exhibited hippocampal-dependent spatial memory impairments at 6 months old. These animals exhibited reference memory deficits as demonstrated by an impaired ability to distinguish a previously unvisited arm in the Y maze forced alternation (FA) test (Fig. 1B). They also had an impaired ability to learn the location of the escape platform in the Morris water maze (MWM) (Fig. 1E-G). These findings are consistent with previous studies showing that early-onset hippocampal-dependent learning and memory deficits can be observed in 5xFAD mice at 6 months old [47-49]. Prolonged MET treatment normalized the spatial memory impairments in 5xFAD mice and had no effect in WT mice (Fig. 1B, E-G). Hippocampal-independent working memory, as assessed by the Y maze spontaneous alternation test, was unchanged in 5xFAD mice or by MET treatment (Supplementary Fig.S1A). Compared to WT-SAL mice, 5xFAD-SAL mice did not show altered anxiety level but had reduced locomotion in the open field test (OFT) (Fig.1C-D). Together, these findings suggest that our cohort of 5xFAD mice did not have prominent anxiety-like phenotypes at 6 months old. Interestingly, prolonged MET treatment significantly increased the time spent in the center of the open field in WT mice and marginally increased the time spent by 5xFAD mice (Fig.1C), showing that MET treatment has anxiolytic effects. Additionally, MET normalized the reduced locomotion in 5xFAD mice (Fig. 1D). Notably, no between-group differences were observed in the total distance traveled in the Y maze FA test or the MWM (Supplementary Fig. 1B-C), suggesting that general locomotor functions and exploratory drive were not affected in 5xFAD-SAL mice. The reduced locomotion observed in the OFT was likely an early sign of emerging anxiety. In addition, prolonged MET administration did not affect the animals’ weight (Supplementary Fig. 1D). Taken together, the results suggest that prolonged MET supplementation has cognitive-enhancing effects in 5xFAD mice.

### Prolonged MET treatment decreases Aβ deposition and microgliosis in the hippocampus of 5xFAD mice

Two hallmarks of AD, Aβ deposition and excessive microglial activation, were quantified in the dorsal hippocampus of 5xFAD mice by immunohistochemistry. Prolonged MET treatment in 5xFAD mice decreased Aβ burden in the dentate gyrus (DG), but not in the CA3 or CA1-2 (Fig. 1H-I). All hippocampal subfields in 5xFAD-SAL mice showed markedly increased coverage of ionizing calcium-binding adapter molecule-positive cells (Iba1+) with activated morphology and clustering (Fig. 1J-K), indicating elevated microglial activation. Microglial activation was also reduced in all hippocampal subfields in MET-treated 5xFAD mice (Fig. 1J-K). These results suggest that MET can alleviate AD neuropathology in the hippocampus of 5xFAD mice.

### Prolonged MET treatment restores DNA methylation dysregulations in the hippocampus of 5xFAD mice

To investigate whether the hippocampal DNA methylation profile is altered in 5xFAD mice and by MET treatment, we quantified global DNA methylation level and transcript levels of DNA methyltransferases (DNMT1, DNMT3a, and DNMT3b) and demethylases (ten-eleven translocation (TET) methylcytosine dioxygenase and growth arrest and DNA-damage-inducible-beta (Gadd45b)). Quantification of global DNA methylation in the dorsal hippocampus by ELISA revealed significantly reduced methylation levels in 5xFAD-SAL mice compared to WT littermates. Notably, MET treatment effectively restored normal methylation levels in 5xFAD mice, while demonstrating no significant effect in WT animals (Fig. 2A). We further quantified global DNA methylation in neuronal cells by immunofluorescence staining of 5-methylcytosine (5mC), which demonstrated consistent but less pronounced methylation differences between groups. Specifically, we found that 5mC expression levels were decreased in the DG granule cell layer (GCL) and CA3 pyramidal cell layer (PCL) in 5xFAD-SAL mice. MET treatment rescued 5mC expression in CA1-2 PCL significantly and in DG GCL marginally (Fig. 2B-E). Deficits in the global DNA methylation level in 5xFAD-SAL mice were likely a result of decreased DNA methylation/demethylation turnover, as we found significantly lower mRNA expressions of both writers and erasers of DNA methylation in these mice. 5xFAD-SAL mice showed decreased hippocampal Dnmt1, Dnmt3a, and Gadd45b mRNA levels (Fig. 2F, H), and marginally decreased Tet1 mRNA level (Fig. 2G). Notably, MET treatment in 5xFAD mice reversed some of these changes, increasing the expression of Dnmt3a, Tet1, and Gadd45b, but not Dnmt1 (Fig. 2F-H).

We next examined DNA methylation at specific gene promoters. We first measured the transcript levels of three genes crucial to memory functions: activity-regulated cytoskeleton-associated protein (Arc), reelin (Reln), and calcineurin (CaN). Both Arc and Reln positively regulate synaptic plasticity [50, 51], whereas CaN, a protein phosphatase, negatively modulates learning and memory [52]. As expected, mRNA levels of Arc and Reln were downregulated in 5xFAD-SAL mice, whereas the mRNA level of CaN was upregulated (Fig. 2I-K). The transcriptional repression of Arc and Reln in 5xFAD mice was accompanied by elevated promoter DNA methylation levels (Fig. 2L-M), whereas the transcriptional upregulation of CaN was accompanied by downregulated CaN promoter DNA methylation level (Fig. 2N). Notably, MET treatment normalized the transcription of the three genes in 5xFAD mice hippocampus by restoring their promoter DNA methylation levels (Fig. 2I-N). These results show that MET can affect DNA methylation at the global level and bidirectionally modulate promoter DNA methylation at the individual gene level.

### Prolonged MET treatment restores protein expression and phosphorylation in MeCP2-CREB-BDNF and CaN-GSK3β pathways in 5xFAD mice

**Figure 3.**
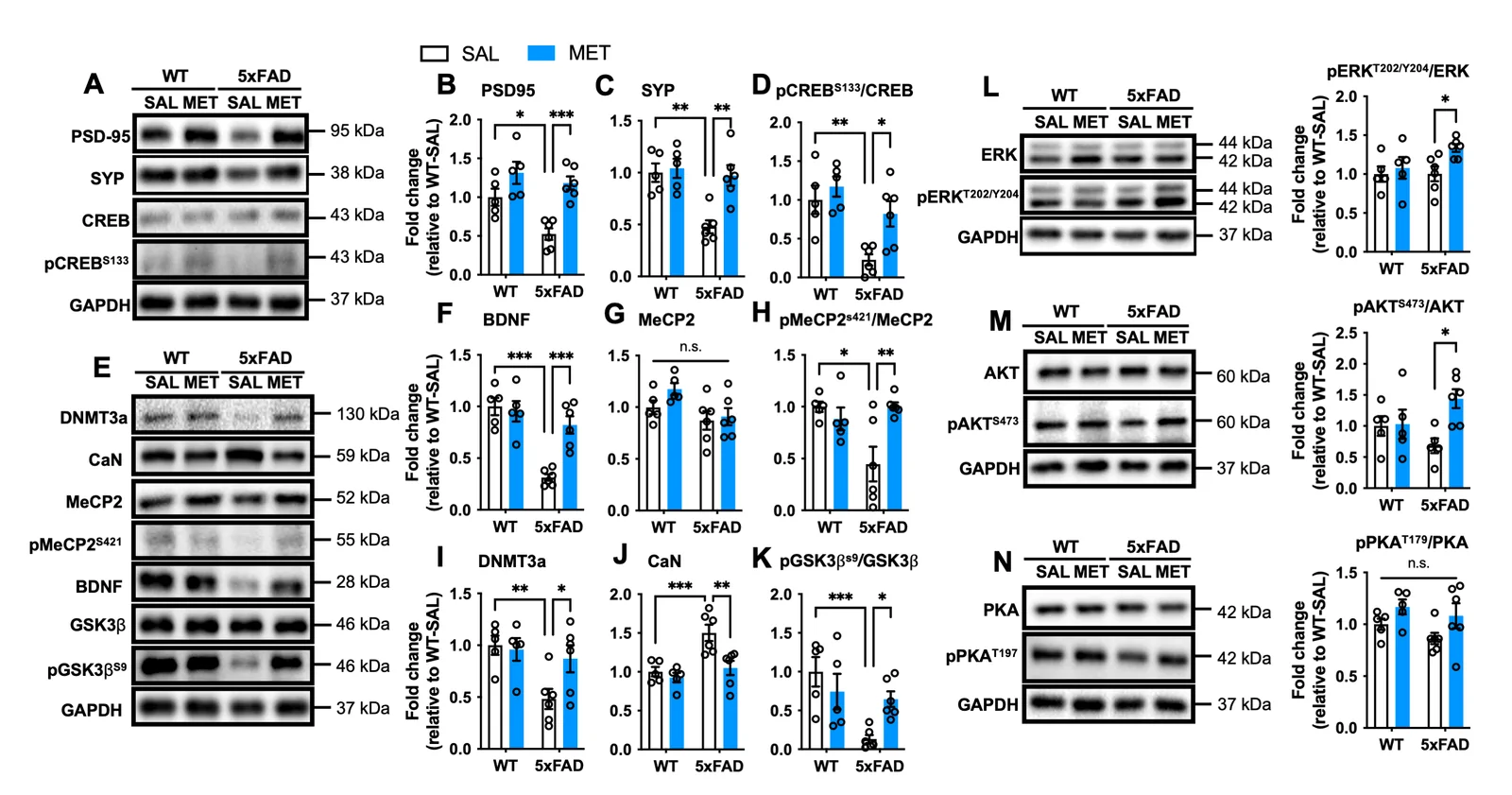
**Protracted MET treatment restores protein expression and phosphorylation in CREB-BDNF and CaN-GSK3β pathways in 5xFAD mice**. (**A**) Representative Western blot images demonstrating the effects of protracted MET treatment on pre- and post-synaptic proteins (SYP and PSD-95), CREB and phosphorylated CREB in WT and 5xFAD mice. (**B-C**) Densitometric analysis of PSD-95 and SYP (two-way ANOVA: PSD95: genotype: *F*_1,18_ = 8.73, *p* = 0.009, treatment: *F*_1,18_ = 22.49, *p* < 0.001; SYP: genotype: *F*_1,18_ = 11.56, *p* = 0.003, treatment: *F*_1,18_ = 9.56, *p* = 0.006, genotype × treatment: *F*_1,18_ = 6.81, *p* = 0.018; Šídák’s post-hoc: WT-SAL vs. 5xFAD-SAL: PSD95: *p* = 0.025, SYP: *p* = 0.003; 5xFAD-SAL vs 5xFAD-MET: PSD95: *p* < 0.001, SYP: *p* = 0.003). (**D**) Densitometric analysis of pCREB^S133^/CREB (two-way ANOVA: PSD95: genotype: *F*_1,18_ = 15.70, *p* = 0.009, treatment: *F*_1,18_ = 7.25, *p* = 0.015; Šídák’s post-hoc: WT-SAL vs. 5xFAD-SAL: *p* = 0.007; 5xFAD-SAL vs 5xFAD-MET: *p* = 0.036). (**E**) Representative Western blot images demonstrating the effects of protracted MET treatment on proteins involved in the CREB-BDNF and CaN-GSK3β pathways in different treatment groups. (**F-K**) Densitometric analysis confirmed altered levels of BDNF, pMeCP2^S421^/MeCP2, DNMT3a, CaN, and pGSK3β^s9^/GSK3β (two-way ANOVA: BDNF: genotype: *F*_1,18_ = 28.10, *p* < 0.001, treatment: *F*_1,18_ = 8.95, *p* = 0.008, genotype × treatment: *F*_1,18_ = 12.87, *p* = 0.002; MeCP2: n.s.; pMeCP2^S421^/MeCP2: genotype × treatment: *F*_1,18_ = 9.54, *p* = 0.006; DNMT3a: genotype: *F*_1,18_ = 7.56, *p* = 0.013, genotype × treatment: *F*_1,18_ = 3.75, *p* = 0.069; CaN: genotype: *F*_1,18_ = 12.98, *p* = 0.002, treatment: *F*_1,18_ = 9.29, *p* = 0.007, genotype × treatment: *F*_1,18_ = 4.71, *p* = 0.044; pGSK3β^s9^/GSK3β: genotype: *F*_1,18_ = 10.62, *p* = 0.004, genotype × treatment: *F*_1,18_ = 6.71, *p* = 0.019; Šídák’s post-hoc: WT-SAL vs. 5xFAD-SAL: BDNF: *p* < 0.001, pMeCP2^S421^/MeCP2: *p* = 0.013, DNMT3a: *p* = 0.004, CaN: *p* < 0.001, and pGSK3β^s9^/GSK3β: *p* < 0.001; 5xFAD-SAL vs 5xFAD-MET: BDNF: *p* < 0.001, pMeCP2^S421^/MeCP2: *p* = 0.008, DNMT3a: *p* = 0.018, CaN: *p* = 0.001, and pGSK3β^s9^/GSK3β: *p* = 0.019). (**L-N**) Representative Western blot images and densitometric analysis of pERK^T202/Y204^/ERK, pAKT^S473^/AKT, and pPKA^T197^/PKA (two-way ANOVA: pERK^T202/Y204^/ERK: treatment: *F*_1,18_ = 4.49, *p* = 0.048; pAKT^S473^/AKT: treatment: *F*_1,18_ = 5.30, *p* = 0.034; pPKA^T197^/PKA: n.s.; Šídák’s post-hoc: 5xFAD-SAL vs 5xFAD-MET: pERK^T202/Y204^/ERK: *p* = 0.021; pAKT^S473^/AKT: *p* = 0.030). Data are presented as means ± s.e.m. N = 5 for WT mice and N = 6 for 5xFAD mice. *p < 0.05, ** *p* < 0.01, ***p < 0.001. n.s., not significant.

As MET restored transcription of synaptic plasticity-regulating genes (Arc, Reln, and CaN) by influencing their promoter DNA methylation, we next investigated whether such modulatory effects were present at the protein level. To this end, we measured protein levels in inter- and intra-cellular signaling pathways regulating synaptic plasticity (Fig. 3).

We first quantified the protein expression of postsynaptic density protein 95 (PSD95), a postsynaptic scaffold protein, and synaptophysin (SYP), a presynaptic vesicle protein, which are both essential for synaptic activity. Changes in the expression of these two proteins have been associated with cognitive decline in AD patients [53, 54]. We found that 5xFAD mice exhibited reduced hippocampal PSD95 and SYP, indicating synaptic degeneration. Treatment with MET restored the expression of PSD95 and SYP in 5xFAD mice to levels comparable to those of WT-SAL mice (Fig. 3A-C), suggesting that MET can potentially restore synaptic structure, promote synaptogenesis, and facilitate synaptic transmission.

It is well established that activation of CREB is required for synaptic plasticity [55]. Phosphorylation at serine 133 (pCREB^S133^) facilitates the transcription of genes, such as BDNF, which are necessary for long-term synaptic plasticity [56]. We found that the pCREB^S133^-BDNF pathway was attenuated in the hippocampus of 5xFAD mice (Fig. 3A, D-F, Supplementary Fig. 2A). Methyl-CpG binding protein 2 (MeCP2) functions as a transcription repressor at BDNF promoter by binding to methylated CpG islands [57]. Activation of pCREB^S133^-BDNF pathway requires phosphorylation of MeCP2 at serine 421 (pMeCP2^S421^), which detaches MeCP2 from BDNF promoter, releasing BDNF from transcription repression [57, 58]. We found that pMeCP2^S421^ was decreased in 5xFAD mice, although total MeCP2 protein expression was unchanged (Fig. 3E, G-H, Supplementary Fig. 2B). Notably, prolonged MET treatment normalized pCREB^S133^, pMeCP2^S421^ and BDNF expressions in 5xFAD mice (Fig. 3A, D-H). In alignment with a decreased global DNA methylation level in 5xFAD mice mentioned above (Fig. 2A-F), we found that 5xFAD mice also exhibited lower hippocampal DNMT3a expression, which was restored by MET treatment (Fig. 3E, I). We next measured protein levels in the CaN-GSK3β pathway, which is thought to negatively influence synaptic plasticity [52] and contribute to Aβ deposition and tau hyperphosphorylation in AD [59]. We found increased CaN protein expression in the hippocampus of 5xFAD mice (Fig. 3E, J), which is consistent with an elevated transcript level, as reported above (Fig. 2K). Notably, CaN has been shown to activate GSK3β via serine 9 dephosphorylation (pGSK3β^S9^) [60]. We found that pGSK3β^S9^ was decreased in 5xFAD-SAL mice compared to WT-SAL mice despite unchanged total protein level (Fig. 3E, K, Supplementary Fig. 2C), suggesting the CaN-GSK3β pathway was hyperactivated in the hippocampus of 5xFAD mice, while MET treatment normalized the expression of CaN and pGSK3β^S9^ (Fig. 3E, K, Supplementary Fig. 2C). Both GSK3β and CREB can be phosphorylated by protein kinases such as extracellular signal-regulated kinase (ERK), protein kinase B (AKT), and protein kinase A (PKA) [61-63].

**Figure 4.**
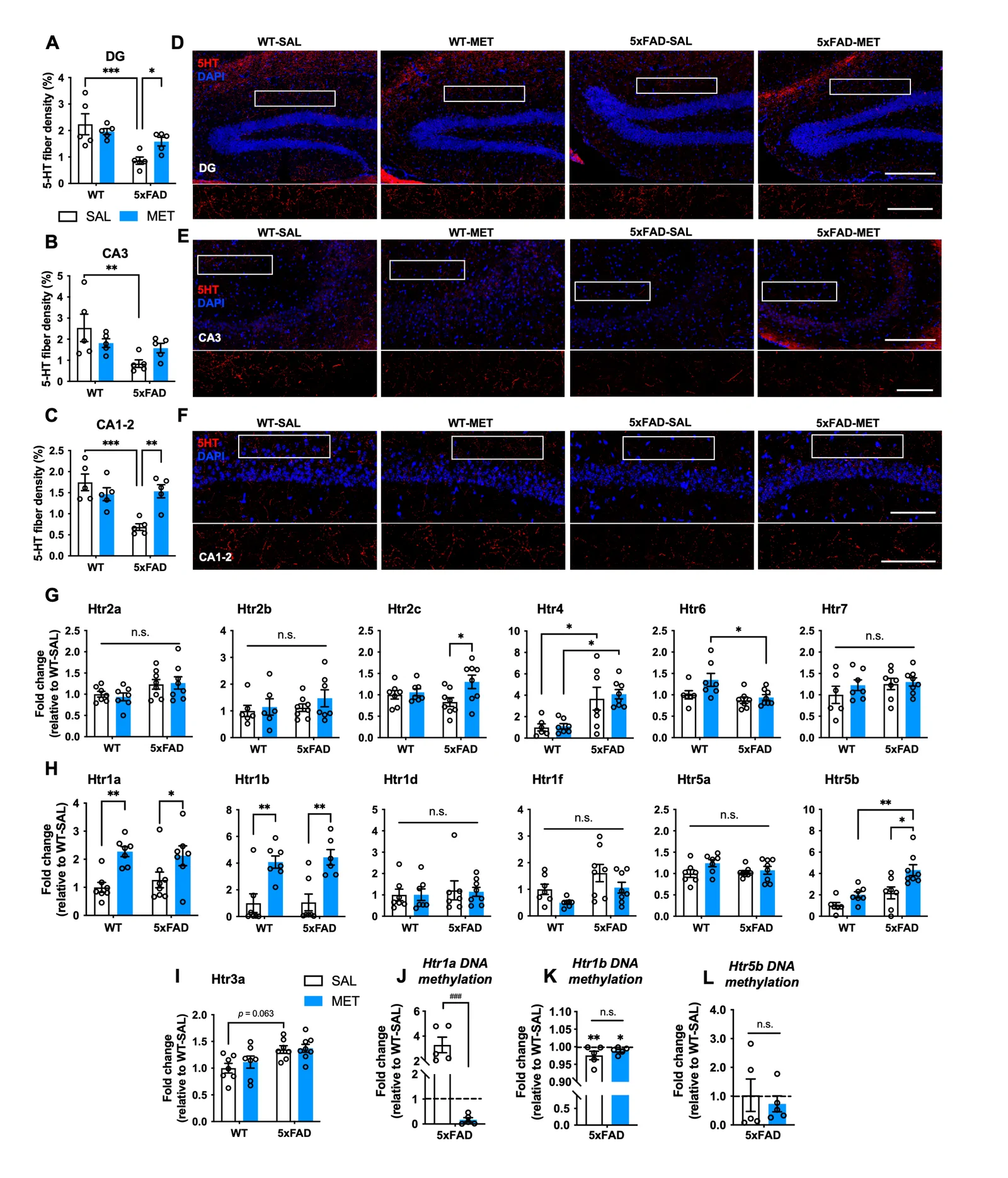
**Protracted MET treatment preserves 5-HT innervation and changed 5-HT receptor expression profile in the hippocampus of 5xFAD mice**. (**A-C**) Immunofluorescence staining was performed to examine 5HT innervation in the DG, CA3, and CA1-2 subfields of dorsal hippocampus (N = 5 per group) (two-way ANOVA: DG: genotype: *F*_1,16_ = 14.32, *p* = 0.002, genotype × treatment: *F*_1,16_ = 4.64, *p* = 0.047; CA3: genotype: *F*_1,16_ = 6.86, *p* = 0.019; CA1-2: genotype: *F*_1,16_ = 10.58, *p* = 0.005, genotype × treatment: *F*_1,16_ = 13.88, *p* = 0.002; Fisher’s LSD post-hoc: WT-SAL vs. 5xFAD-SAL: DG: *p* < 0.001; CA3: *p* = 0.005; CA1-2: *p* < 0.001; 5xFAD-SAL vs 5xFAD-MET: DG: *p* = 0.043; CA1-2: *p* = 0.001). (**D-F**) Representative immunofluorescence images of 5HT (red) and DAPI (blue) in the DG, CA3 and CA1-2 subregions of the dorsal hippocampus (scale bars: DG 65 μm, CA3 40 μm, CA1-2 25 μm, insets 15 μm). (**G-H**) Transcript levels of Gα_s_/Gα_q_ coupled 5HT receptors (G), Gα_i/o_ coupled 5HT receptors (H), and Htr3a (I) in the dorsal hippocampus were quantified by RT-qPCR (N = 6-8 per group) (two-way ANOVA: Htr2a: n.s.; Htr2b: n.s.; Htr2c: treatment: *F*_1,26_ = 5.14, *p* = 0.032; Htr4: genotype: *F*_1,24_ = 20.81, *p* < 0.001; Htr6: genotype: *F*_1,24_ = 7.07, *p* = 0.014; Htr7: n.s.; Htr1a: treatment: *F*_1,25_ = 16.70, *p* < 0.001; Htr1b: treatment: *F*_1,23_ = 28.79, *p* < 0.001; Htr1d: n.s.; Htr1f: n.s.; Htr5a: n.s.; Htr5b: genotype: *F*_1,24_ = 15.23, *p* < 0.001; treatment: *F*_1,24_ = 12.39, *p* = 0.002; Htr3a: genotype: *F*_1,25_ = 11.81, *p* = 0.002; Šídák’s post-hoc: WT-SAL vs 5xFAD-SAL: Htr4: *p* = 0.042; Htr3a: *p* = 0.063; WT-MET vs 5xFAD-MET: Htr4: *p* = 0.010; Htr6: *p* = 0.038; Htr5b: *p* = 0.005; WT-SAL vs WT-MET: Htr1a: *p* = 0.002; Htr1b: *p* = 0.007; 5xFAD-SAL vs 5xFAD-MET: Htr2c: *p* = 0.039; Htr1a: *p* = 0.026; Htr1b: *p* = 0.004; Htr5b: *p* = 0.011). (**J-L**) Promoter DNA methylation levels of genes which showed more than 2-fold change by MET in qPCR analysis (Htr1a, Htr1b, and Htr5b) were quantified by methylation-specific qPCR (N = 5 per group) (Kruskal-Wallis H tests: Htr1a: *H*(2) = 12.50, *p* < 0.001, Htr1b: *H*(2) = 9.52, *p* = 0.002, Htr5b: n.s.; Dunn’s multiple comparison tests: WT-SAL vs 5xFAD-SAL: Htr1a: adjusted *p* = 0.077, Htr1b: adjusted *p* = 0.005, WT-SAL vs 5xFAD-MET: Htr1a: adjusted *p* = 0.077, Htr1b: adjusted *p* = 0.013, 5xFAD-SAL vs 5xFAD-MET: Htr1a: adjusted *p* < 0.001, Htr1b: n.s.). Data are presented as means ± s.e.m. *p < 0.05, ** *p* < 0.01, ***p < 0.001. n.s., not significant.

We found the total protein levels of these kinases were not changed in 5xFAD mice or by MET treatment (Fig. 3L-N, Supplementary Fig. 2D-F). However, MET treatment increased the relative proportion of phosphorylated ERK and phosphorylated AKT compared to their respective total protein levels (Fig. 3L-N). Together, these results showed that MET treatment can rescue synaptic functions in 5xFAD mice by restoring the expression of synaptic proteins and by modulating the phosphorylation of proteins in MeCP2-CREB-BDNF and CaN-GSK3β pathways.

### Prolonged MET treatment restores 5-HT innervation and alters 5-HT receptor expression profile in the hippocampus of 5xFAD mice

We investigated whether hippocampal 5-HT neurotransmission was altered in 5xFAD mice and by MET treatment. We observed that 5-HT projection fiber density was reduced in all subregions of the dorsal hippocampus in 5xFAD mice (Fig. 4A-F). Hippocampal 5-HT fiber degeneration likely contributes to the cognitive impairments in 5xFAD mice [64]. Prolonged MET treatment restored 5-HT innervation in all hippocampal subregions, with the most prominent effects in CA1-2 (Fig. 4A-F), suggesting that maintaining normal levels of DNA methylation protects the integrity of the 5-HT projection fibers in the hippocampus of 5xFAD mice.

Changes in the transcript levels of certain 5-HT receptors were also observed in the hippocampus of 5xFAD mice. Among the six Gα_s_/Gα_q_-coupled 5-HT receptors, only the mRNA level of Htr4 was increased in 5xFAD mice (Fig. 4G). Treatment with MET had little effect on the transcription of hippocampal Gα_s_/Gα_q_-coupled 5-HT receptors, except for a slight increase in the expression of Htr2c in 5xFAD mice (Fig. 4G). Transcript levels of the six Gα_i/o_-coupled 5-HT receptors were all comparable between WT-SAL and 5xFAD-SAL mice (Fig. 4H). Treatment with MET significantly upregulated Htr1a and Htr1b mRNA levels in WT and 5xFAD mice, and upregulated Htr5b mRNA levels in 5xFAD mice (Fig. 4H). The transcript level of Htr3a, the only 5-HT receptor that is a ligand-gated cation channel, was slightly upregulated in the hippocampus of 5xFAD mice (Fig. 4I), which was not altered by MET treatment (Fig. 4I). We next examined whether the transcriptional changes in Htr1a, Htr1b, and Htr5b induced by MET treatment were accompanied by promoter DNA methylation alterations in 5xFAD mice. We found that the MET-induced transcriptional upregulation of Htr1a in 5xFAD mice was accompanied by a significant reduction in promoter DNA methylation (Fig. 4J). Interestingly, while no differences in Htr1a or Htr1b transcript levels were observed between WT-SAL and 5xFAD-SAL mice (Fig. 4H), promoter DNA methylation patterns of Htr1a and Htr1b differed between the two genotypes. Htr1a methylation was marginally higher in 5xFAD-SAL mice (Fig. 4J), whereas Htr1b methylation was lower in 5xFAD mice compared to WT mice, irrespective of SAL or MET treatment (Fig. 4K). These findings suggest that basal DNA methylation levels at gene promoters can vary between WT and 5xFAD mice, and that the regulatory impact of promoter methylation on transcription of a certain gene may differ between genotypes. Promoter DNA methylation level of Htr5b were both unchanged in 5xFAD-SAL mice compared to WT-SAL mice and were unaffected by MET treatment (Fig. 4L). Together, these results suggest that 5-HT neurotransmission is altered in 5xFAD mice, which can be modulated by MET treatment.

### Dnmt3a knockdown diminishes the pro-cognitive effects of prolonged MET treatment in 5xFAD mice

**Figure 5.**
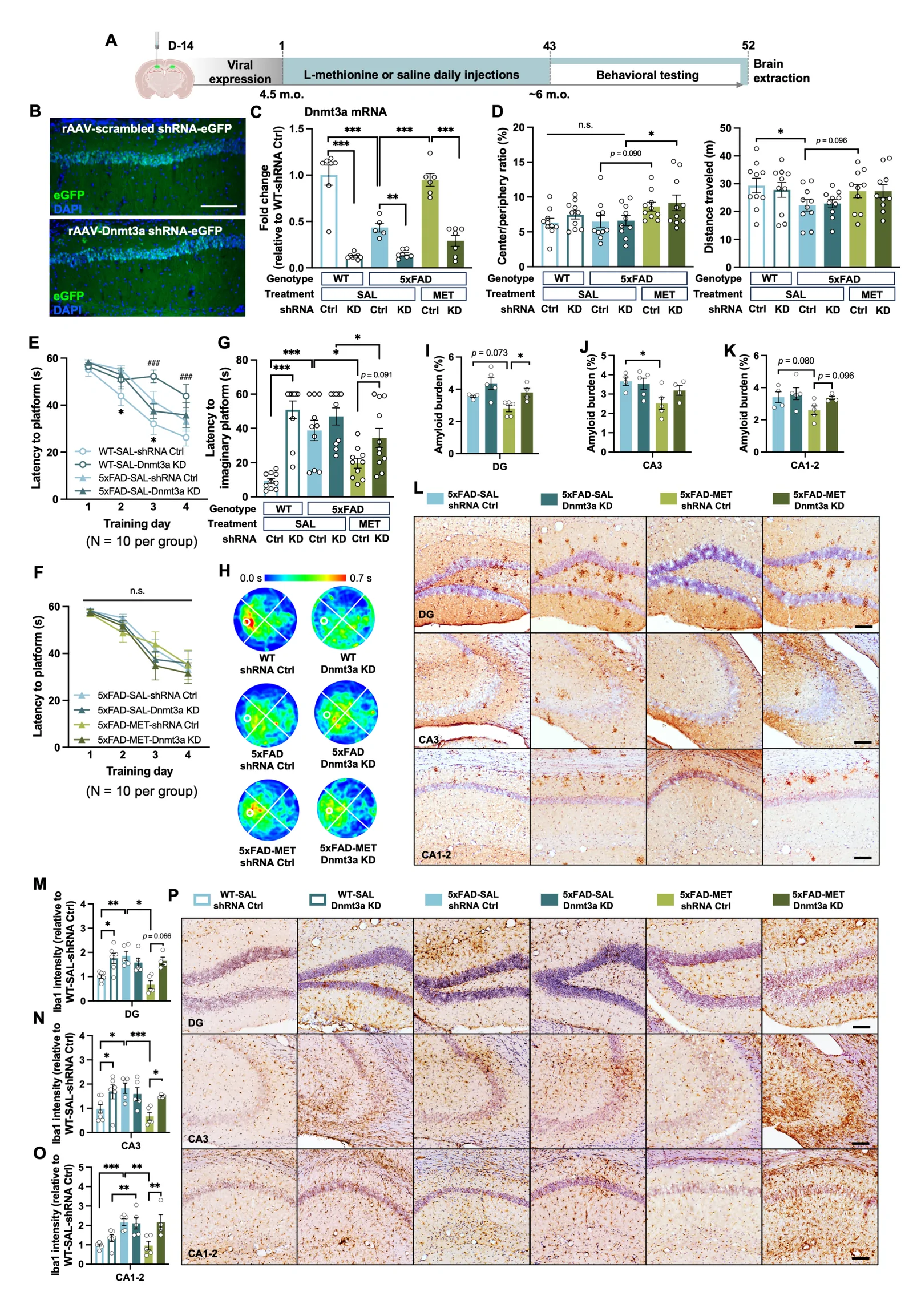
**Dnmt3a knockdown partially cancelled the pro-cognitive effects of protracted MET treatment in 5xFAD mice**. (**A**) WT or 5xFAD mice receiving bilateral dorsal CA1-2 stereotaxic injections of rAAVs containing eGFP tag plus either Dnmt3a shRNA or scrambled control shRNA were treated with SAL or MET for 1.5 months before and throughout behavioral tests. Mice were 4.5 months old (m.o.) at the start of SAL or MET treatment and roughly 6 m.o. at the start of behavioral tests. (**B**) rAAV delivery to the dorsal CA1-2 was confirmed by eGFP (green) labelling of the pyramidal cell layer of CA1-2 (scale bar 150 μm). (**C**) Knockdown of Dnmt3a in the dorsal hippocampus was confirmed by RT-qPCR analysis (N = 6-8 per group) (two-way ANOVA with Šídák’s post-hoc: WT-SAL-shRNA Ctrl vs WT-SAL-Dnmt3a KD: *p* < 0.001; 5xFAD-SAL-shRNA Ctrl vs 5xFAD-SAL-Dnmt3a KD: *p* = 0.025; 5xFAD-MET-shRNA Ctrl vs 5xFAD-MET-Dnmt3a KD: *p* < 0.001; WT-SAL-shRNA Ctrl vs 5xFAD-SAL-shRNA Ctrl: *p* < 0.001; 5xFAD-SAL-shRNA Ctrl vs 5xFAD-MET-shRNA Ctr: *p* < 0.001). (**D**) Ratio of time spent in the center versus the periphery (left) and the total distance travelled (right) in the open field test (N = 10 per group) (two-way ANOVA with Šídák’s post-hoc: center/periphery ratio: 5xFAD-SAL-shRNA Ctrl vs 5xFAD-MET-shRNA Ctrl: *p* = 0.090; 5xFAD-SAL-Dnmt3a KD vs 5xFAD-MET-Dnmt3a KD: *p* = 0.044; distance travelled: WT-SAL-shRNA Ctrl vs 5xFAD-SAL-shRNA Ctrl: *p* = 0.034; 5xFAD-SAL-shRNA Ctrl vs 5xFAD-MET-shRNA Ctr: *p* = 0.096). (**E-G**) Learning performances in the MWM was measured by the latency to reach the platform location during training days (E-F) and on probe test (G) (N = 10 per group) (E: three-way ANOVA with Fisher’s LSD post-hoc: WT-SAL-shRNA Ctrl vs 5xFAD-SAL-shRNA Ctrl: *p* = 0.022 on day 2 and *p* = 0.050 on day 3, indicated by *; WT-SAL-shRNA Ctrl vs WT-SAL-Dnmt3a KD: *p* < 0.001 on day 3 and 4, indicated by ###; F: three-way ANOVA with Fisher’s LSD post-hoc: n.s.; G: two-way ANOVA with Šídák’s post-hoc: WT-SAL-shRNA Ctrl vs WT-SAL-Dnmt3a KD: *p* < 0.001; WT-SAL-shRNA Ctrl vs 5xFAD-SAL-shRNA Ctrl: *p* < 0.001; 5xFAD-SAL-shRNA Ctrl vs 5xFAD-MET-shRNA Ctrl: *p* = 0.010; 5xFAD-SAL-Dnmt3a KD vs 5xFAD-MET-Dnmt3a KD: *p* = 0.038; 5xFAD-MET-shRNA Ctrl vs 5xFAD-MET-Dnmt3a KD: *p* = 0.091). (**H**) Cumulative swim tracks visualized by heat maps by each group during MWM probe test. (**I-K**) Analysis of amyloid-β burden (4g8) in the DG, CA3 and CA1-2 of dorsal hippocampus in 5xFAD mice (N = 4-5 per group) (two-way ANOVA with Šídák’s post-hoc: 5xFAD-SAL-shRNA Ctrl vs 5xFAD-MET-shRNA Ctrl: DG: *p* = 0.073; CA3: *p* = 0.014; CA1-2: *p* = 0.080; 5xFAD-MET-shRNA Ctrl vs 5xFAD-MET-Dnmt3a KD: DG: *p* = 0.023; CA1-2: *p* = 0.096). (**L**) Representative images of 4G8 IHC staining in the dorsal hippocampus (scale bars:100 μm). (**M-O**) Analysis of microglia activation (Iba1) in the DG, CA3 and CA1-2 of dorsal hippocampus in WT or 5xFAD mice (N = 4-7 per group) (two-way ANOVA with Šídák’s post-hoc: WT-SAL-shRNA Ctrl vs 5xFAD-SAL-shRNA Ctrl: DG: *p* = 0.009; CA3: *p* = 0.019; CA1-2: *p* < 0.001; 5xFAD-SAL-shRNA Ctrl vs 5xFAD-MET-shRNA Ctrl: DG: *p* = 0.015; CA3: *p* < 0.001; CA1-2: *p* = 0.005; WT-SAL-shRNA Ctrl vs WT-SAL-Dnmt3a KD: DG: *p* = 0.016; CA3: *p* = 0.039; WT-SAL-Dnmt3a KD vs 5xFAD-SAL-Dnmt3a KD: CA1-2: *p* = 0.008; 5xFAD-MET-shRNA Ctrl vs 5xFAD-MET Dnmt3a KD: DG: *p* = 0.066; CA3: *p* = 0.012; CA1-2: *p* = 0.008). (**P**) Representative images of Iba1 IHC staining in the dorsal hippocampus (scale bars:100 μm). Data are presented as means ± s.e.m. *p < 0.05, ** *p* < 0.01, ***p < 0.001. n.s., not significant.

We found that DNMT3a, but not DNMT3b, was downregulated in the hippocampus of 5xFAD mice, and this was restored by MET treatment (Fig. 2F, 3I). This led us to speculate that DNMT3a is responsible for mediating the effects of MET. To this end, we conducted hippocampal-specific Dnmt3a knockdown (KD) to investigate the mechanism of the pro-cognitive effects of MET in 5xFAD mice (Fig. 5A). Short hairpin RNA (shRNA) targeting Dnmt3a or control shRNA (Supplementary Fig. 3A) was delivered to hippocampal CA1-2 via recombinant adeno-associated virus (rAAV) infection 2 weeks before the start of SAL/MET treatment (Fig. 5A-B). The KD effects of Dnmt3a shRNA were confirmed by RT-qPCR in WT-SAL, 5xFAD-SAL, and 5xFAD-MET mice (Fig. 5C). Dnmt3b mRNA levels did not differ between WT and 5xFAD mice and were not affected by SAL/MET treatment or rAAV infusion (Supplementary Fig. 3B). In the OFT, Dnmt3a KD did not have any effect on anxiety or locomotion in WT or 5xFAD mice (Fig. 5D). In the MWM, MET treatment restored the impaired spatial learning and memory in 5xFAD mice (Fig. 5G).

Dnmt3a KD markedly impaired spatial learning and memory in WT mice during both training and probe test (Fig. 5E, G-H, Supplementary Fig. 3C). However, Dnmt3a KD did not further impair spatial memory in 5xFAD mice (Fig. 5F-H). 5xFAD-SAL-shRNA Ctrl mice already exhibited severely impaired spatial memory during the probe test, as evidenced by their prolonged latency (38.8 ± 18.8 s) in reaching the imaginary platform location. Although the 5xFAD-SAL-Dnmt3a shRNA group displayed an even greater latency (47.0 ± 15.8 s), the already profound deficit in the control group likely limited the detectability of further deterioration, resulting in a non-significant intergroup difference. Notably, Dnmt3a KD diminished the memory enhancing effects of MET (Fig. 5F-H). Overall, locomotor abilities and exploratory drive were not affected by Dnmt3a KD, as the total distance traveled in the MWM probe test did not differ between groups (Supplementary Fig. 3D). These results suggest that the pro-cognitive effects of MET in 5xFAD mice are at least partially mediated by Dnmt3a.

### Dnmt3a knockdown abolishes the Aβ-reducing and anti-microgliosis effects of prolonged MET treatment in 5xFAD mice

To investigate the role of Dnmt3a in AD-related pathology, we next assessed the impact of Dnmt3a KD on Aβ deposition and microglial activation in the dorsal hippocampus. In 5xFAD mice, Dnmt3a KD did not exacerbate Aβ accumulation further, consistent with the severe baseline pathology in this model. However, it significantly attenuated the Aβ-reducing effects of MET, particularly in the dentate gyrus (DG), with a marginal but observable effect in CA3 and CA1-2 subregions (Fig. 5I-L). Similarly, while Dnmt3a KD markedly enhanced microglial activation in WT mice (Fig. 5M-P), it did not further increase microgliosis in 5xFAD mice, likely due to their already pronounced neuroinflammatory state. Notably, Dnmt3a KD impaired the ability of MET to suppress microglial activation in 5xFAD mice, diminishing its anti-microgliosis effects across all hippocampal subfields (Fig. 5M-P). The absence of additional Aβ deposition or microglial hyperactivation in 5xFAD-SAL-Dnmt3a shRNA mice suggests that pre-existing severe pathology in 5xFAD mice leaves little room for further deterioration. Nevertheless, importantly, these findings indicate that MET’s Aβ-lowering and anti-inflammatory effects in 5xFAD mice are, at least partially, dependent on Dnmt3a activity.

**Figure 6.**
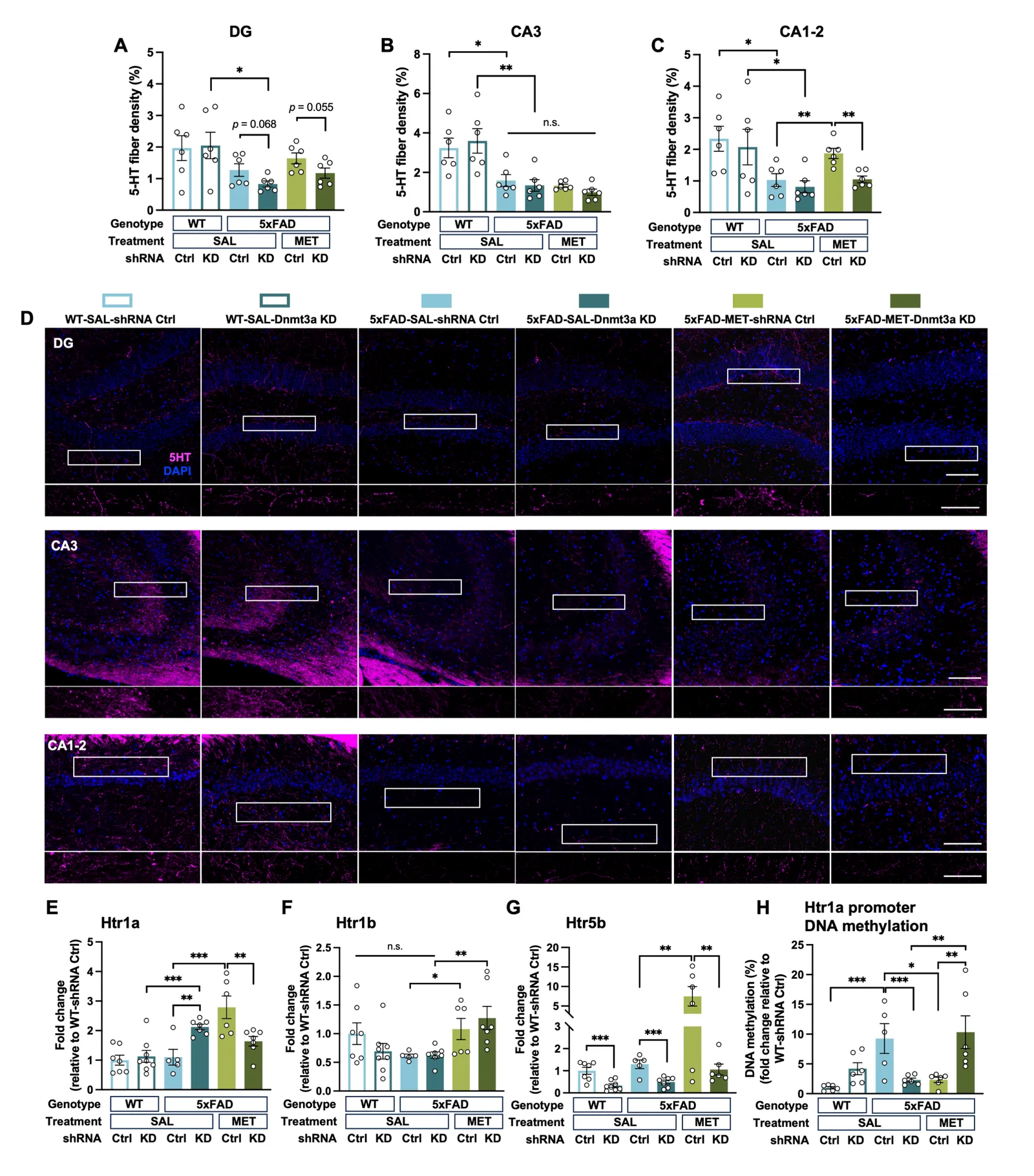
**Dnmt3a knockdown counteracted the 5HT fibre-restoring effects of protracted MET treatment in 5xFAD mice**. (**A-C**) Effects of MET and Dnmt3a knockdown (KD) on 5HT innervation in the dorsal hippocampus were examined by immunofluorescence staining (N = 6 per group) (two-way ANOVA with Šídák’s post-hoc: WT-SAL-shRNA Ctrl vs 5xFAD-SAL-shRNA Ctrl: CA3: *p* = 0.017; CA1-2: *p* = 0.022; WT-SAL-Dnmt3a KD vs 5xFAD-SAL-Dnmt3a KD: DG: *p* = 0.011; CA3: *p* = 0.002; CA1-2: *p* = 0.026; 5xFAD-SAL-shRNA Ctrl vs 5xFAD-SAL-Dnmt3a KD: DG: *p* = 0.068; 5xFAD-SAL-shRNA Ctrl vs 5xFAD-MET-shRNA Ctrl: CA1-2: *p* = 0.002; 5xFAD-MET-shRNA Ctrl vs 5xFAD-MET-Dnmt3a KD: DG: *p* = 0.055; CA1-2: *p* = 0.003). (**D**) Representative images of 5HT (magenta) and DAPI (blue) in the DG, CA3 and CA1-2 subregions of the dorsal hippocampus (scale bars: DG and CA3 20 μm, CA1-2 25 μm, insets 15 μm). (**E-G**) Transcript levels of 5HT1a, 5-HT1b, and 5-HT5b receptors in the dorsal hippocampus were quantified by RT-qPCR (N = 5-8 per group) (two-way ANOVA with Šídák’s post-hoc: WT-SAL-shRNA Ctrl vs WT-SAL-Dnmt3a KD: Htr5b: *p* < 0.001; WT-SAL-Dnmt3a KD vs 5xFAD-SAL-Dnmt3a KD: Htr1a: *p* < 0.001; 5xFAD-SAL-shRNA Ctrl vs 5xFAD-SAL-Dnmt3a KD: Htr1a: *p* = 0.001; Htr5b: *p* < 0.001; 5xFAD-SAL-shRNA Ctrl vs 5xFAD-MET-ShRNA Ctrl: Htr1a: *p* < 0.001; Htr1b: *p* = 0.049; Htr5b: *p* = 0.003; 5xFAD-SAL-Dnmt3a KD vs 5xFAD-MET-Dnmt3a KD: Htr1b: *p* = 0.004; 5xFAD-MET-shRNA Ctrl vs 5xFAD-MET-Dnmt3a KD: Htr1a: *p* = 0.002; Htr5b: *p* = 0.002). (**H**) Promoter DNA methylation levels of of Htr1a were quantified by methylation-specific qPCR (N = 5-6 per group) (two-way ANOVA with Šídák’s post-hoc: WT-SAL-shRNA Ctrl vs 5xFAD-SAL-shRNA Ctrl: *p* < 0.001; 5xFAD-SAL-shRNA Ctrl vs 5xFAD-SAL-Dnmt3a KD: *p* < 0.001; 5xFAD-SAL-shRNA Ctrl vs 5xFAD-MET-shRNA Ctrl: *p* = 0.016; 5xFAD-SAL-Dnmt3a KD vs 5xFAD-MET-Dnmt3a KD: *p* = 0.005; 5xFAD-MET-shRNA Ctrl vs 5xFAD-MET-Dnmt3a KD: *p* = 0.005). Data are presented as means ± s.e.m. *p < 0.05, ** *p* < 0.01, ***p < 0.001. n.s., not significant.

### Dnmt3a knockdown counteracts the 5-HT fiber-restoring effects of prolonged MET treatment in 5xFAD mice

We next examined the effects of Dnmt3a KD on 5-HT neurotransmission in the dorsal hippocampus. We observed 5-HT projection fiber degeneration in 5xFAD mice (Fig. 6A-D), while MET treatment restored 5-HT innervation in the CA1-2 (Fig. 6C-D). Dnmt3a KD did not change 5-HT fiber density in WT-SAL mice (Fig. 6A-D). It marginally decreased 5-HT fiber density in the DG in 5xFAD-SAL mice and diminished the 5-HT fiber-restoring effects of MET in the DG and CA1-2 of 5xFAD-MET mice (Fig. 6A, C-D). We previously observed that MET markedly increased Htr1a and Htr1b mRNA levels in both WT and 5xFAD mice, and Htr5b mRNA levels specifically in 5xFAD mice (Fig. 4H). Hence, we next investigated if Dnmt3a KD would abolish these effects. Dnmt3a KD did not change Htr1a mRNA levels in WT mice, but it increased Htr1a mRNA levels in 5xFAD mice (Fig. 6E). Furthermore, Htr1a mRNA levels were reduced by Dnmt3a KD in 5xFAD-MET mice (Fig.6E). Transcript levels of Htr1b were not altered by Dnmt3a KD in any group (Fig. 6F). Dnmt3a KD reduced Htr5b mRNA levels in WT-SAL, 5xFAD-SAL, and 5xFAD-MET mice (Fig. 6G). We previously found that Htr1a transcription upregulation in 5xFAD-MET mice was accompanied by promoter DNA demethylation (Fig. 4J), which was also confirmed in this experiment (Fig. 6H). Notably, Htr1a transcription upregulation in 5xFAD-SAL-Dnmt3a KD mice was accompanied by decreased promoter DNA methylation, whereas Htr1a transcription downregulation in 5xFAD-MET-Dnmt3a KD mice was accompanied by increased promoter DNA methylation (Fig. 6H). This supports the idea that Htr1a transcription is under the direct control of promoter DNA methylation. Together, these results suggest that MET-induced 5-HT innervation rescue and transcriptional upregulation of Htr1a and Htr5b in 5xFAD mice require Dnmt3a, whereas MET-induced transcriptional upregulation of Htr1b is likely mediated by other pathways. Additionally, our results suggest that Dnmt3a can regulate Htr1a and Htr5b transcription independently of MET, as Dnmt3a-KD alone altered the transcript levels of these genes.

### Dnmt3a overexpression impairs spatial memory and increases anxiety-like behaviors in WT and 5xFAD mice

**Figure 7.**
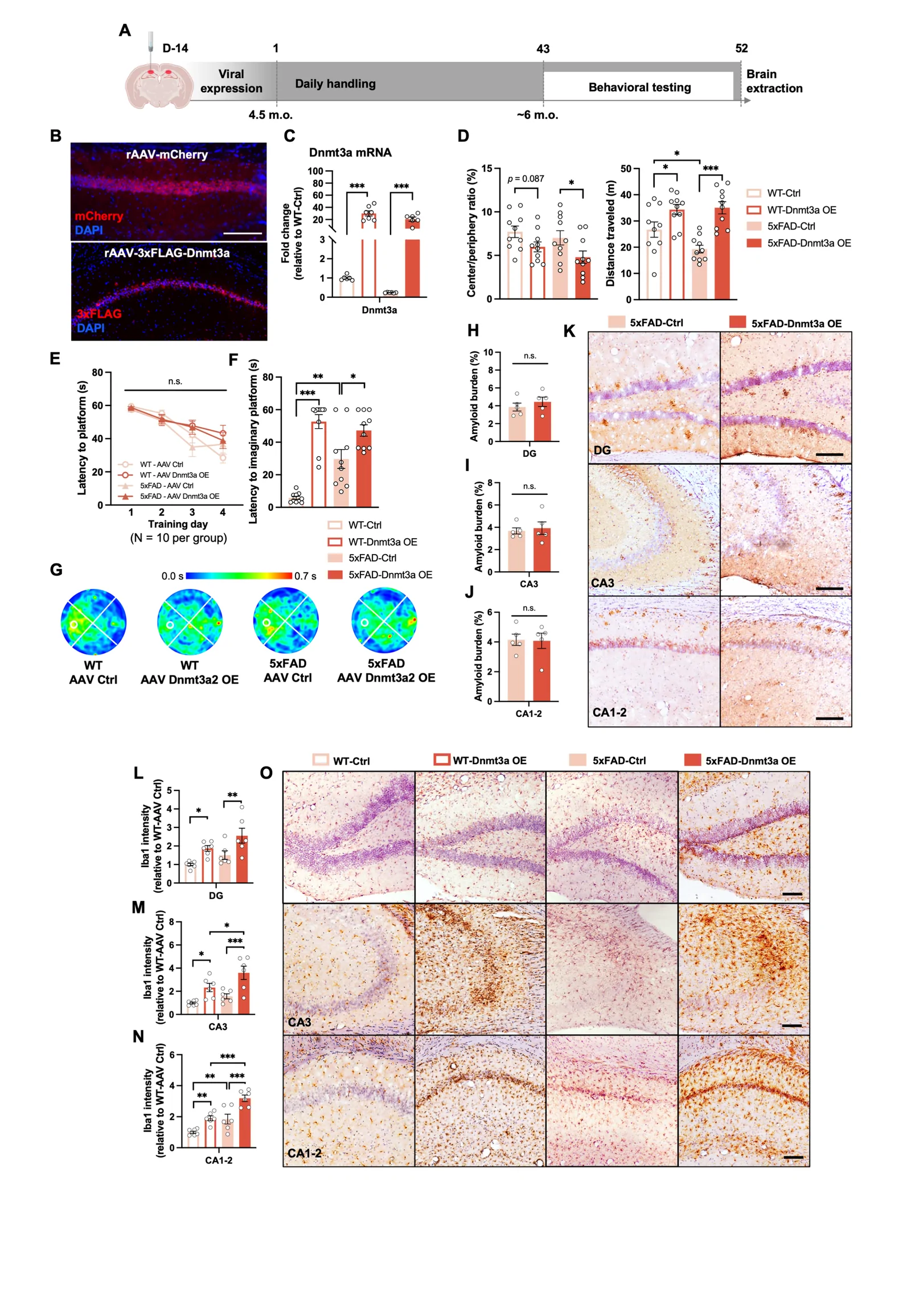
**Dnmt3a overexpression impairs spatial memory and increases anxiety-like behaviors in WT and 5xFAD mice**. (**A**) WT or 5xFAD mice receiving bilateral dorsal CA1-2 stereotaxic injections of rAAVs containing 3xFLAG tagged Dnmt3a or only a mCherry tag were handled daily by experimenters for 1.5 months before behavioral tests. Mice were roughly 6 months old (m.o.) at the start of behavioral tests. (**B**) rAAV delivery to the dorsal CA1-2 was confirmed by mCherry labelling or 3xFLAG expression (red) in the pyramidal cell layer of CA1-2 (scale bar 150 μm). (**C**) Overexpression of Dnmt3a in the dorsal hippocampus was confirmed by RT-qPCR analysis (N = 6-7 per group) (two-way ANOVA with Šídák’s post-hoc: WT-Ctrl vs WT-Dnmt3a OE: *p* < 0.001; 5xFAD-Ctrl vs 5xFAD-Dnmt3a OE: *p* < 0.001). (**D**) Ratio of time spent in the center versus the periphery (left) and the total distance travelled (right) in the open field test (N = 10 per group) (two-way ANOVA with Šídák’s post-hoc: center/periphery ratio: WT-Ctrl vs WT-Dnmt3a OE: *p* = 0.087; 5xFAD-Ctrl vs 5xFAD-Dnmt3a OE: *p* = 0.030; distance travelled: WT-Ctrl vs WT-Dnmt3a OE: *p* = 0.021; 5xFAD-Ctrl vs 5xFAD-Dnmt3a OE: *p* = 0.030; WT-Ctrl vs 5xFAD-Ctrl: *p* = 0.023). (**E-F**) Learning performances in the MWM was measured by the latency to reach the platform location during training days (E) and on probe test (F) (N = 10 per group) (E: three-way ANOVA: n.s.; F: two-way ANOVA with Šídák’s post-hoc: WT-Ctrl vs WT-Dnmt3a OE: *p* < 0.001; 5xFAD-Ctrl vs 5xFAD-Dnmt3a OE: *p* = 0.024; WT-Ctrl vs 5xFAD-Ctrl: *p* = 0.001). (**G**) Cumulative swim tracks visualized by heat maps by each group during MWM probe test. (**H-J**) Analysis of amyloid-β burden (4g8) in the DG, CA3 and CA1-2 of dorsal hippocampus in 5xFAD mice (N = 4-5 per group) (Man-Whitney U tests: *p*-values > 0.050). (**K**) Representative images of 4G8 IHC staining in the dorsal hippocampus (scale bars:100 μm). (**L-N**) Expression of microglial marker Iba1 in the DG, CA3 and CA1-2 subregions of dorsal hippocampus in WT or 5xFAD mice (N = 4-5 per group) (two-way ANOVA with Šídák’s post-hoc: WT-Ctrl vs WT-Dnmt3a OE: DG: *p* = 0.015; CA3: *p* = 0.011; CA1-2: *p* = 0.004; 5xFAD-Ctrl vs 5xFAD-Dnmt3a OE: DG: *p* = 0.006; CA3: *p* < 0.001; CA1-2: *p* < 0.001; WT-Ctrl vs 5xFAD-Ctrl: CA1-2: *p* = 0.006; WT-Dnmt3a OE vs 5xFAD-Dnmt3a OE: CA3: *p* = 0.019; CA1-2: *p* < 0.001). (**O**) Representative images of Iba1 IHC staining in the dorsal hippocampus (scale bars:100 μm). Data are presented as means ± s.e.m. *p < 0.05, ** *p* < 0.01, ***p < 0.001. n.s., not significant.

Given that the beneficial effects of prolonged MET treatment in 5xFAD mice were mediated by Dnmt3a, we hypothesized that direct hippocampal Dnmt3a overexpression (OE) might similarly enhance cognitive function. To test this possibility, we investigated the potential cognitive-enhancing effects of Dnmt3a overexpression (OE) in the hippocampus. We delivered 3xFLAG-tagged Dnmt3a OE rAAVs or control rAAVs (Supplementary Fig. 4A) to hippocampal CA1-2 in WT and 5xFAD mice (Fig. 7A-B). These mice did not receive any MET injections but were handled daily by the experimenters. The RT-qPCR analysis showed Dnmt3a-OE rAAVs significantly increased the mRNA levels of Dnmt3a in both WT and 5xFAD mice (Fig. 7C). Dnmt3b mRNA levels did not differ between WT and 5xFAD mice and were not affected by rAAV infusion (Supplementary Fig. 4B). Contrary to expectations, we observed that Dnmt3a OE increased anxiety and caused hyperactivity in both groups in the OFT (Fig. 7D), and significantly impaired spatial learning and memory in the MWM (Fig. 7E-G, Supplementary Fig. 4C). Overall, locomotor abilities and exploratory drive were not affected by Dnmt3a OE, as the total distance traveled in MWM probe test did not differ between groups (Supplementary Fig. 4D). These results suggest that increasing Dnmt3a availability in the hippocampus has anxiogenic and memory-impairing effects.

### Dnmt3a overexpression does not affect Aβ deposition but increases microglial activation in WT and 5xFAD mice

We next examined the effects of Dnmt3a OE on Aβ deposition and microglial activation in the dorsal hippocampus. Dnmt3a OE did not alter Aβ deposition across hippocampal subregions in 5xFAD mice (Fig. 7H-K). Notably, Dnmt3a OE significantly enhanced microglial activation in both 5xFAD and WT mice, further exacerbating the already elevated microglial response in 5xFAD animals (Fig. 7L-O).

### Dnmt3a overexpression increases hippocampal 5-HT fiber density in WT and 5xFAD mice

**Figure 8.**
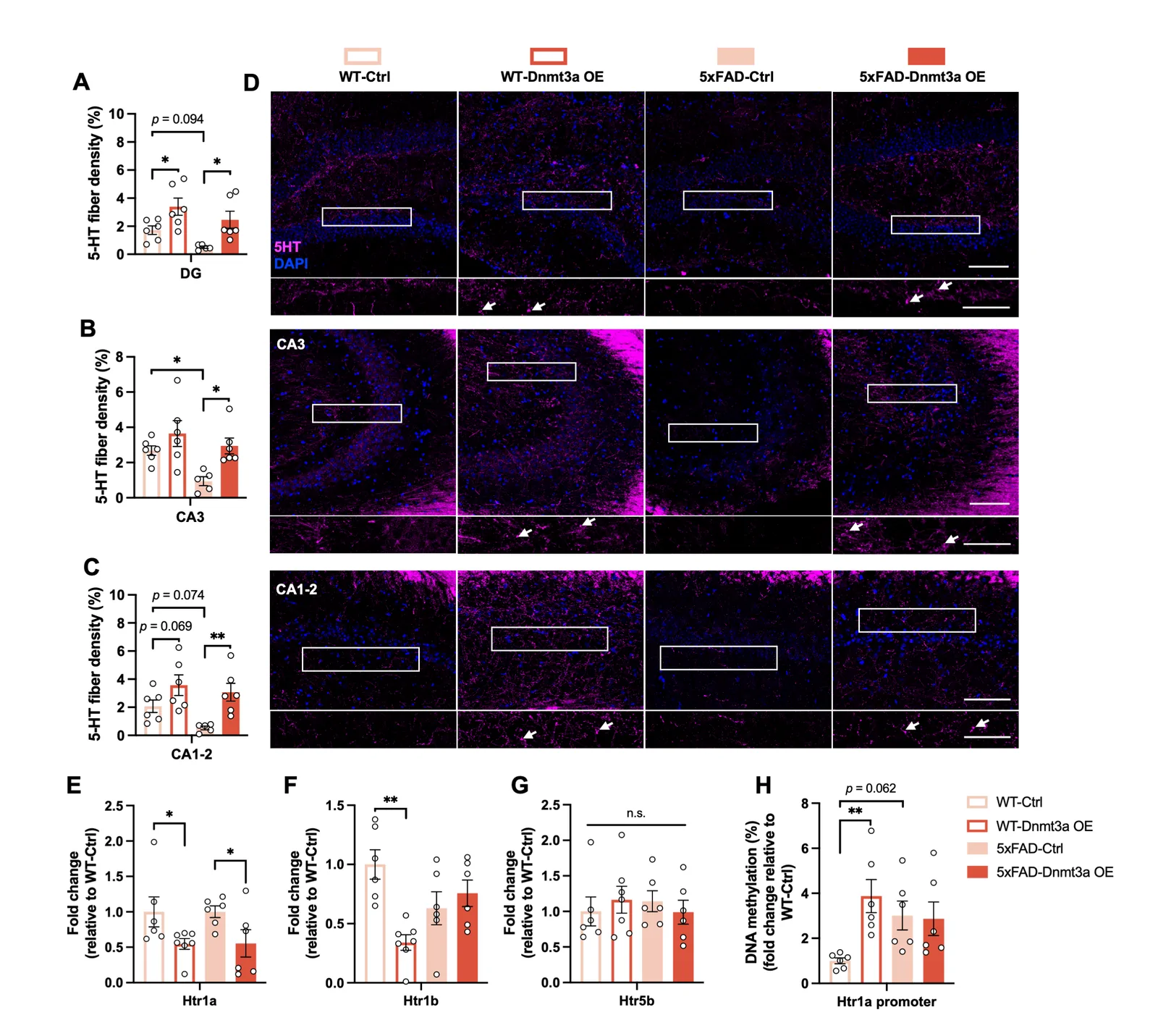
**Dnmt3a overexpression increased hippocampal 5HT fibre density in WT and 5xFAD mice**. (**A-C**) Effects Dnmt3a overexpression (OE) on 5HT innervation in the dorsal hippocampus were examined by immunofluorescence staining (N = 5-7 per group) (two-way ANOVA with Šídák’s post-hoc: WT-Ctrl vs WT-Dnmt3a OE: DG: *p* = 0.021; CA1-2: *p* = 0.069; WT-Ctrl vs 5xFAD-Ctr: CA3: *p* = 0.023; CA1-2: *p* = 0.074; 5xFAD-Ctrl vs 5xFAD-Dnmt3a OE: DG: *p* = 0.011; CA3: *p* = 0.010; CA1-2: *p* = 0.006). (**D**) Representative images of 5HT (magenta) and DAPI (blue) in the DG, CA3 and CA1-2 subregions of the dorsal hippocampus (scale bars: DG and CA3 20 μm, CA1-2 25 μm, insets 15 μm). Arrows point to swollen varicosities on 5-HT fibers. (**E-G**) Transcript levels of 5HT1a, 5-HT1b, and 5-HT5b receptors in the dorsal hippocampus were quantified by RT-qPCR (N = 6-7 per group) (two-way ANOVA with Šídák’s post-hoc: WT-Ctrl vs WT-Dnmt3a OE: Htr1a: *p* = 0.040; Htr1b: *p* = 0.002; 5xFAD-Ctrl vs 5xFAD-Dnmt3a OE: Htr1a: *p* = 0.050). (**H**) Promoter DNA methylation levels of Htr1a were quantified by methylation-specific qPCR (N = 6 per group) (two-way ANOVA with Šídák’s post-hoc: WT-Ctrl vs WT-Dnmt3a OE: *p* = 0.004, WT-Ctrl vs 5xFAD-Ctrl: *p* = 0.062). Data are presented as means ± s.e.m. *p < 0.05, ** *p* < 0.01, ***p < 0.001. n.s., not significant.

We next investigated the consequences of hippocampal Dnmt3a OE on 5-HT neurotransmission. Consistent with our previous observations, 5xFAD mice exhibited significant 5-HT denervation in the dorsal hippocampus (Fig. 8A-D). Intriguingly, Dnmt3a OE enhanced 5-HT fiber density in both genotypes, with a more pronounced effect in 5xFAD mice (Fig. 8A-D). However, these fibers displayed abnormal morphology characterized by swollen varicosities (Fig. 8D, arrows) - a well-established hallmark of axonal degeneration [65, 66]. This suggests that while Dnmt3a OE promotes 5-HT fiber outgrowth, it may simultaneously compromise their structural integrity and functionality. Next, we examined if Dnmt3a OE affected the mRNA levels of Htr1a, Htr1b, and Htr5b. Dnmt3a OE resulted in decreased Htr1a mRNA levels in both WT and 5xFAD mice (Fig. 8E) and increased Htr1b mRNA levels only in WT mice (Fig. 8F), but Htr5b mRNA levels were not affected (Fig. 8G). Additionally, Dnmt3a OE increased promoter DNA methylation of Htr1a only in WT mice (Fig. 8H).

### Dnmt3a knockdown/overexpression impairs LTP and paired-pulse responses at CA3-CA1 synapses in WT mice

**Figure 9.**
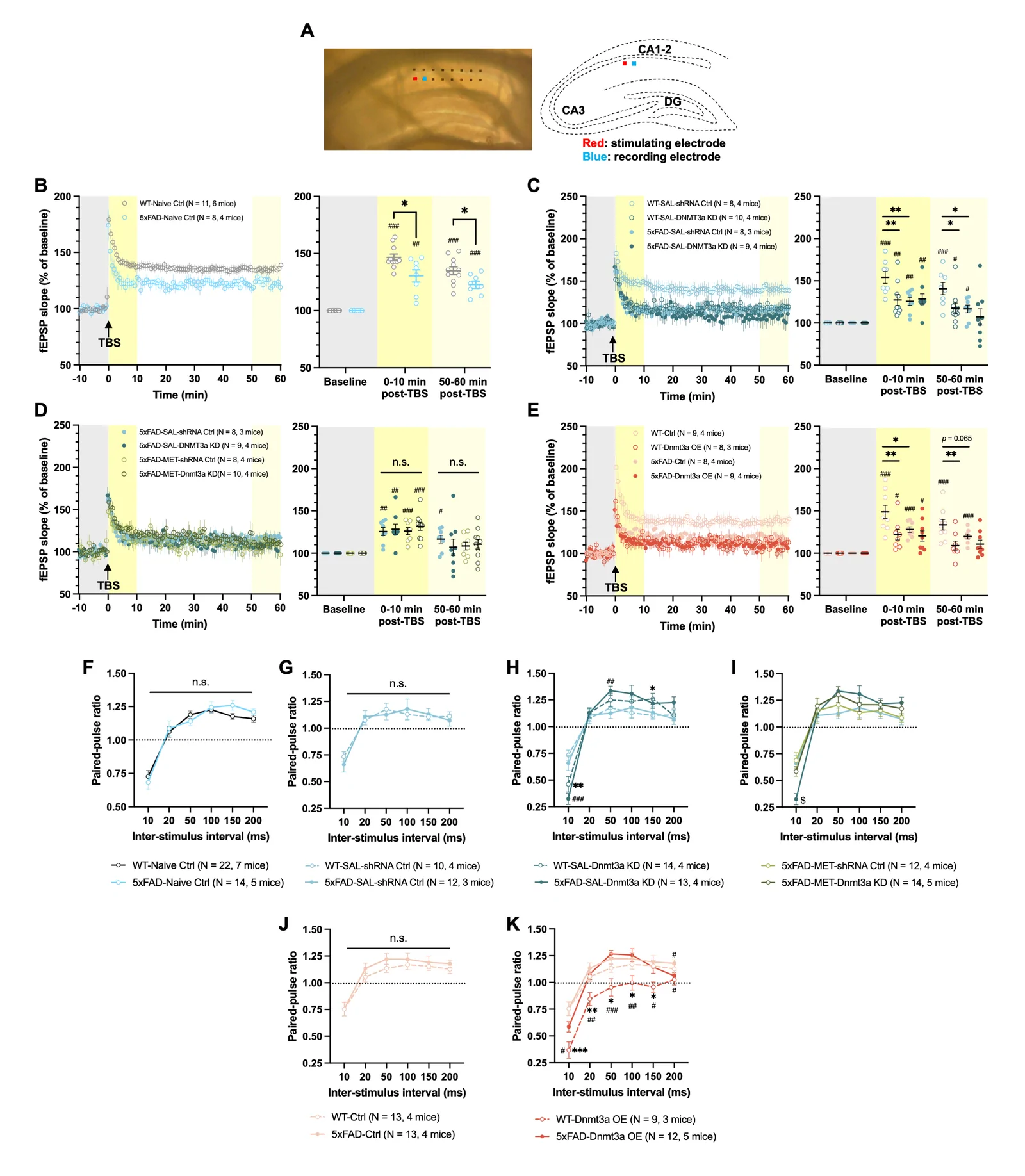
**Dnmt3a knockdown or overexpression impaired LTP and paired pulse responses at CA3-CA1 synapse in WT mice**. (**A**) Photo and schematics showing the placement of stimulating and recording electrodes to target the CA3-CA1 Schaffer collateral pathway. (**B-E**) Graphs showing fEPSP slopes before and after TBS (left) and averaged fEPSP slopes (right) during baseline (averaged over the first 10 min), 0-10 min after TBS, and 50-60 min after TBS of naïve WT and 5xFAD mice (B), WT and 5xFAD mice receiving SAL injections and either control shRNA or Dnmt3a KD shRNA (C), 5xFAD mice receiving either SAL or MET injections and either control shRNA or Dnmt3a KD shRNA (D), and WT and 5xFAD mice receiving either control rAAVs or Dnmt3a overexpression (OE) rAAVs (E). Between-group differences were tested by two-way ANOVA followed by Šídák’s post-hoc tests and are indicated on the graphs by *p < 0.050, **p < 0.010 and ***p < 0.001. Differences in fEPSP slopes before and after TBS of each group were tested by paired-samples *t*-tests and significant differences are indicated by ^#^*p* < 0.050, ^##^*p* < 0.010 and ^###^*p* < 0.001. (**F-I**) Paired pulse ratios (PPR, slope of the second fEPSP divided by slope of the first fEPSP) of naïve WT and 5xFAD mice (F), WT and 5xFAD mice receiving SAL injections and control shRNA (G-H), and 5xFAD mice receiving either SAL or MET injections and either control shRNA or Dnmt3a KD shRNA (H-I) at interstimulus intervals (ISI) of 10, 20, 50, 100, 150, and 200 ms. Between-group differences were tested by two-way ANOVA followed by Šídák’s post-hoc tests and are indicated on the graphs by *p < 0.050, **p < 0.010 and ***p < 0.001 relative to WT-shRNA Ctrl group, # for differences compared to 5xFAD-shRNA Ctrl group, and $ for differences compared to 5xFAD-MET-Dnmt3a KD group. 3-7 mice per group. (**J-K**) Paired pulse ratios (PPR, slope of the second fEPSP divided by slope of the first fEPSP) of WT and 5xFAD mice receiving either control rAAVs or Dnmt3a OE rAAVs at interstimulus intervals (ISI) of 10, 20, 50, 100, 150, and 200 ms. Between-group differences were tested by two-way ANOVA followed by Šídák’s post-hoc tests and are indicated on the graphs by *p < 0.050, **p < 0.010 and ***p < 0.001 relative to WT-Ctrl group, # for differences compared to 5xFAD-Dnmt3a OE group. 3-5 mice per group. n.s., not significant. At least 2 hippocampal slides per mouse were tested and analysed. The total numbers of slides (N) per group are indicated in legends. Data shown as mean ± s.e.m.

We observed that prolonged MET treatment rescued the synaptic degeneration and dysregulation of synaptic plasticity-regulating signaling pathways in the hippocampus of 5xFAD mice. Hence, we next investigated if MET also modulated neurotransmission at CA3-CA1 synapses. We measured long-term potentiation (LTP) and paired-pulse responses (PPR) at CA3-CA1 synapses in hippocampal explant slices (Fig. 9A). Measurements were first made in naïve WT and naïve 5xFAD mice (WT-Naïve Ctrl and 5xFAD-Naïve Ctrl) to establish the baseline synaptic transmission profiles of the two genotypes. Theta burst stimulation (TBS)-induced field excitatory postsynaptic potential (fEPSP) slopes were observed in both groups in the early (0 - 10 min) and late (50 - 60 min) phases post stimulation (Fig. 9B). Consistent with previous studies [67], we observed decreased LTP in 5xFAD mice compared to their WT littermates (Fig. 9B), indicating impaired postsynaptic plasticity at CA3-CA1 synapses. We found that both Dnmt3a OE and KD impaired CA3-CA1 LTP in the early and late phases in WT mice (Fig. 9C, E), while Dnmt3a OE and KD further impaired CA3-CA1 LTP in the late phase in 5xFAD mice, but the magnitude of change was relatively small compared to that in WT mice (Fig. 9C, E). The difference in the magnitude of the impairment between WT and 5xFAD mice is likely due to a “floor effect” in 5xFAD mice, where postsynaptic plasticity is already weak and is insensitive to further disruption by Dnmt3a OE or KD. More detailed electrophysiology experiments are needed to test this hypothesis. Nevertheless, our results show that perturbed Dnmt3a levels impaired LTP. Interestingly, MET treatment did not rescue the impaired LTP in 5xFAD mice (Fig. 9D).

Presynaptic plasticity was probed by measuring the paired-pulse ratio of fEPSPs elicited by two consecutive stimuli. Paired-pulse depression (PPD) was observed at an interstimulus interval (ISI) of 10 ms and paired-pulse facilitation (PPF) was observed at all other ISI levels for all groups except for WT-Dnmt3a OE group (Fig. 9F-K). We found that neither the 5xFAD genotype nor MET treatment had any effect on PPR (Fig. 9F-G, I-J, Supplementary Fig. 5A-C), suggesting that presynaptic plasticity is preserved in 5xFAD mice and unaffected by MET. Dnmt3a KD caused exaggerated PPD at an ISI of 10 ms and modestly enhanced PPF at ISIs above 20 ms in both WT and 5xFAD mice (Fig. 9H, Supplementary Fig. 5B). Dnmt3a OE attenuated PPF at an ISI of 200 ms in 5xFAD mice and completely abolished PPF in WT mice. Dnmt3a OE also changed the response from facilitation to depression at an ISI of 20 ms and caused exaggerated PPD at an ISI of 10 ms in WT mice (Fig. 9K, Supplementary Fig. 5C). Taken together, these results suggest that the MET-induced pro-cognitive effects in 5xFAD mice were independent of synaptic transmission at CA3-CA1 synapses. Perturbations in Dnmt3a levels, either an increase or decrease, impaired both pre- and post-synaptic plasticity at CA3-CA1 synapses.

## DISCUSSION

In the present study, we showed that dysregulation of hippocampal DNA methylation contributes to the neuropathology in 5xFAD mice. We observed impaired spatial memory in 5xFAD mice, which was accompanied by global DNA hypomethylation and aberrant DNA methylation changes at specific gene promoters. Mechanistically, we identified reduced expression of key methylation regulators - including DNMTs (DNMT1, DNMT3a) and demethylases (TET1, Gadd45b) - suggesting impaired methylation/demethylation dynamics in 5xFAD hippocampus (Fig. 1, Fig. 2A-H). These changes led to bidirectional promoter methylation alterations: hypermethylation at Arc and Reln promoters (associated with transcriptional suppression) and hypomethylation at the CaN promoter (linked to transcriptional activation) (Fig.2I-N). Our results add to the growing body of literature that supports DNA methylation dysregulation as a key factor contributing to AD pathogenesis [68]. This highlights the importance of studying both the mechanisms underlying cerebral DNA hypomethylation in the 5xFAD model and therapeutic approaches to correct these pathological epigenetic modifications. Such research holds promises for developing targeted interventions applicable to the subset of AD patients exhibiting global DNA hypomethylation, potentially enabling precision medicine approaches for this distinct patient population.

One important finding of the present study is that methyl supplementation was effective in restoring hippocampal functions in 5xFAD mice. Prolonged treatment with MET improved spatial memory and exerted a wide range of beneficial effects at the molecular level in the hippocampus of 5xFAD mice. MET treatment increased global DNA methylation and upregulated the expression of DNMT3a, TET1, and Gadd45b (Fig.2 A-H), suggesting the restoration of DNA methylation/demethylation turnover. Additionally, MET reversed the aberrant changes in DNA methylation at Arc, Reln, and CaN promoters and normalized their transcript expression (Fig. 2I-N), suggesting that MET can bidirectionally regulate DNA methylation at specific gene promoters.

Prolonged MET treatment also alleviated AD-associated neuropathologies in the hippocampus of 5xFAD mice. First, MET decreased Aβ plaque load in the DG (Fig. 1H-I), suggesting that Aβ accumulation could be reduced by targeting DNA methylation processes. This finding is in line with previous studies that showed the expression of genes involved in the amyloidogenic pathway, such as APP, BACE1 and PSEN1, was regulated by DNA methylation (reviewed by [4]). Second, MET reduced microglial activation in all hippocampal subfields (Fig. 1J-K). It has been shown that genes regulating neuroinflammation and microglial activation, such as interleukin (IL)-1β, IL-6, and caspase-4, are regulated by DNA methylation [69, 70]. Our findings suggested that MET has anti-inflammatory properties in the hippocampus, possibly through influencing DNA methylation profiles of inflammatory genes. Third, we found that MET rescued the expression of synaptic proteins PSD95 and SYP, and normalized protein expression and phosphorylation in MeCP2-CREB-BDNF and CaN-GSK3β pathways, which are crucial pathways that regulate synaptic plasticity and memory [52, 56-58].

The brain 5-HT system plays a crucial role in modulating memory and emotional processes [71]. Both clinical and preclinical studies have demonstrated significant alterations in hippocampal 5-HT neurotransmission in AD [72-76]. In the current study, we examined hippocampal 5-HT integrity in 5xFAD mice and evaluated the therapeutic potential of MET intervention. Our results revealed consistent 5-HT fiber degeneration throughout the hippocampal formation of 5xFAD mice (Fig.4, Fig. 6, Fig.8), mirroring observations from post-mortem AD brains showing reduced hippocampal 5-HT levels [75]. Our findings also align with previous reports of diminished 5-HT projections in CA1 of 5xFAD mice [64, 72]. Notably, two other transgenic models of AD, 3xTg-AD and hAPP_Sw,Ind_, were found to have either unaltered or increased 5-HT fiber density in the hippocampus [73, 74]. The discrepancy in the findings between 5xFAD mice and the other two AD models can be explained by differences in pathology severity and progression. Compared to other transgenic models, the 5xFAD model is more aggressive as it incorporates more familial AD mutations that lead to earlier and more severe presentation of pathologies, such as Aβ plaque deposition and neuroinflammation [77]. Notably, the 5xFAD model closely mimics disease conditions in human AD. Moreover, transcriptomic changes in 5xFAD mice more closely resemble those in human AD than in other transgenic models [78]. Hence, we believe that the 5xFAD model is more suitable for studying changes in 5-HT innervation in AD. We found that MET induced memory enhancements in 5xFAD mice were accompanied by the rescue of 5-HT innervation in the hippocampus, suggesting that maintaining normal levels of DNA methylation protects the integrity of 5-HT projection fibers in the hippocampus of 5xFAD mice. However, the cellular and molecular mechanisms underlying such effects require further investigation.

Interestingly, mRNA levels of 5-HT receptors were mostly unaltered in the hippocampus of 5xFAD mice (Fig. 4), except that Htr3a and Htr4 were upregulated. In the hippocampus, 5-HT3A receptors are expressed exclusively by cholecystokinin-containing interneurons [79]. Activation of 5-HT3A receptors has been linked to gamma oscillation interference [80], hence, the upregulation of 5-HT3A receptors in the hippocampus of 5xFAD mice might contribute to the memory impairments by disrupting the electrical activity of the local inhibitory interneuron network. Existing findings regarding brain 5-HT4 receptor expression in AD patients are mixed. Studies have reported increases, decreases, and lack of change in 5-HT4 receptor expression in AD patients [81-83]. Many preclinical studies have also shown that 5-HT4 receptor activation has cognitive-enhancing effects [84-86]. The increase in 5-HT4 transcription observed in 5xFAD-SAL mice might be a compensatory response to the decreased availability of 5-HT caused by fiber degeneration. Notably, in the current study, the expression levels of 5-HT1A, 5-HT1B, and 5-HT2A receptors were similar between WT and 5xFAD mice, which contradicts existing evidence showing decreased levels of these receptors in the hippocampus and cortical regions in AD patients [87-89] and AD transgenic mice [90, 91]. This discrepancy may be attributed to the fact that the present study measured mRNA levels whereas existing evidence of decreased expression is based on protein levels. Together, this could suggest that the decreased protein expression of these receptors under AD conditions is likely due to disruptions in translation or accelerated degradation, while transcription remains unaffected.

Treatment with MET significantly upregulated mRNA levels of 5-HT1A and 5-HT1B receptors in both WT and 5xFAD mice, and mRNA levels of 5-HT5B receptors specifically in 5xFAD mice. The functional implications of these changes are particularly noteworthy given the established roles of these receptors in cognitive processes. 5-HT1A receptors play a well-documented but complex role in hippocampal-dependent memory, with both agonism and antagonism producing variable effects on cognition (see reviews [92, 93]). Few studies have focused on 5-HT1B receptors. Existing reports show that they exert predominantly inhibitory effects on learning and memory, as evidenced by studies showing memory impairments following receptor activation and improvements with blockade in rodents [94-96]. Notably, 5-HT5B receptors, while absent in humans [97], are expressed broadly in rodent brains and contribute significantly to neurodevelopment and cognitive function [98-100]. Nevertheless, the therapeutic relevance of these findings is underscored by the high expression of both 5-HT1A and 5-HT1B receptors in the human hippocampus [101, 102], suggesting that MET-mediated modulation of these receptors may have translational potential. Our results indicate that the cognitive benefits of MET in 5xFAD mice likely involve enhanced hippocampal 5-HT neuromodulation through these receptor subtypes, although the precise downstream mechanisms remain to be elucidated.

We found that MET treatment reduced Htr1a promoter methylation in 5xFAD mice (Fig.4J), suggesting that MET increases Htr1a transcription by downregulating Htr1a promoter DNA methylation. Interestingly, compared to WT-SAL mice, 5xFAD-SAL mice exhibited higher promoter methylation of Htr1a, but this did not correspond to lower transcription (Fig.4J), which is contradictory to the conventional notion that DNA methylation decreases transcription. It is possible that mice with different genetic backgrounds have different basal levels of DNA methylation and gene transcription. Alternatively, it is possible that changes in other epigenetic modification processes counteracted the effects of increased DNA methylation. For example, Htr1a repression is mediated by Deaf-1 [103] and Freud-1 [104] binding at the Htr1a promoter, and thus disruption of these processes under neuropsychiatric conditions [105] could release Htr1a from transcription repression. Promoter DNA methylation levels of Htr1b and Htr5b were unchanged by MET treatment. This lack of change could be a result of simultaneous upregulation of methylation writer Dnmt3a and erasers Tet1 and Gadd45b by MET. Another possibility is that Htr1b and Htr5b promoter CpG island methylation levels were not affected by AD conditions or MET treatment. Hence, MET-induced transcription enhancement of Htr1b and Htr5b are likely mediated by other molecular pathways.

We subsequently investigated the role of hippocampal DNMT3a in AD pathogenesis and in the memory-enhancing effects induced by MET. We found that maintaining physiological DNMT3a levels is essential for normal cognitive function, as both KD and OE impaired memory in WT and 5xFAD mice. Notably, DNMT3a KD abolished MET's beneficial effects on memory, Aβ deposition, neuroinflammation, and serotonergic signaling (Htr1a/Htr5b expression) in 5xFAD mice (Fig. 5-6), suggesting that Dnmt3a mediates the beneficial effects of methyl supplementation in AD mice. In WT mice, Dnmt3a KD impaired spatial memory and increased microglia activation. These results suggest that Dnmt3a is necessary for normal memory functions and the maintenance of microglia quiescence in healthy individuals. Dnmt3a KD did not change 5-HT innervation in the hippocampus of WT-SAL and 5xFAD-SAL mice, suggesting that it is not required for the maintenance of 5-HT innervation in WT and 5xFAD mice.

Dnmt3a OE impaired spatial memory and induced heightened anxiety in both WT and 5xFAD mice. The impairment of memory contradicts previous findings that found overexpression of Dnmt3a2 (one of the two isoforms of Dnmt3a) rescued the impaired memory in aged mice [106]. One possible explanation is that adult WT and 5xFAD mice (6 months old, used in the current study) react differently to Dnmt3a OE compared to aged WT mice (18 months old, used in [106]). Our findings suggest that higher-than-needed levels of Dnmt3a impair hippocampal functions in healthy adult WT mice. Other studies have also reported adverse effects following Dnmt3a OE in healthy mice. Dnmt3a OE in the nucleus accumbens of mice increased depressive behaviors [45] and rendered male mice more susceptible to stress [107]. It was intriguing to find that Dnmt3a OE in 5xFAD mice also showed adverse effects, because like aged mice [106], 5xFAD mice also have lower basal levels of Dnmt3a. It is possible that under diseased conditions, Dnmt3a OE fails to perform correct functions, leading to erroneous DNA methylation and further disrupting gene transcription. Dnmt3a OE did not change hippocampal Aβ plaque burden in 5xFAD mice, suggesting that the behavioral effects and other cellular or molecular effects of Dnmt3a OE were not secondary to changes in Aβ burden. Notably, Dnmt3a OE markedly increased microglial activation in the hippocampus of both WT and 5xFAD mice. Hence, the memory impairments and anxiety in these mice were possibly caused by increased neuroinflammation. Dnmt3a OE also caused a marked increase in hippocampal 5-HT fiber density (Fig. 8). However, these fibers were possibly dysfunctional as they contained many swollen varicosities that are characteristic of degenerating 5-HT axons [65, 66]. Moreover, it has been previously reported that excessive 5-HT causes damage in the body. Excess 5-HT in the body induced by drug use is known to cause serotonin syndrome, a life-threatening condition [108]. One CNS symptom of serotonin syndrome is agitation, which was also observed in the current study. Additionally, one study showed that the optogenetic activation of the raphe-hippocampus circuit promoted anxiety-like behaviors in female mice [109]. Dnmt3a OE also decreased 5-HT1a receptor transcript levels, which could be a compensatory response to increased 5-HT neurotransmission due to increased 5-HT fiber innervation. Activation of Htr1a receptors has been shown to be anxiolytic [110], hence, decreased 5-HT1a receptor expression might contribute to heightened anxiety in Dnmt3a OE mice.

Results from our electrophysiology experiments revealed that perturbations in Dnmt3a levels impaired pre- and post-synaptic plasticity at CA3-CA1 synapses (Fig. 9). While 5xFAD mice exhibited impaired LTP, their paired-pulse responses were comparable to those of WT mice. Interestingly, MET treatment did not rescue CA3-CA1 LTP in 5xFAD mice (Fig. 9). Both Dnmt3a OE and KD impaired LTP in the early and late phases post-TBS. Long-term maintenance of LTP involves gene transcription [111], which requires dynamic and reversible *de novo* DNA methylation and demethylation [112, 113]. Therefore, Dnmt3a OE and KD likely impaired late-phase LTP by disrupting activity-induced *de novo* DNA methylation. The induction of LTP and early-phase LTP primarily depend on local changes in postsynaptic dendrites, such as Ca2+/CaM-dependent protein kinase II activation, AMPA receptor insertion into the postsynaptic membrane, and the reorganization of postsynaptic densities [114, 115]. As these processes do not require gene transcription, it is unclear how changes in Dnmt3a levels influence these processes, which should be unaffected by changes in DNA methylation. Here, we propose two possibilities. First, basal expression levels of postsynaptic proteins and mRNA necessary for early LTP may be decreased in animals after Dnmt3a OE and KD, leading to less excitable postsynaptic dendrites. Second, as induction and maintenance of LTP requires mitochondrial functions in postsynaptic dendrites [116], and Dnmt3a is expressed in mitochondria and regulates mitochondrial DNA methylation [117, 118], Dnmt3a OE and KD may influence early-phase LTP via regulating mitochondrial DNA methylation in postsynaptic dendrites.

Early electrophysiology studies have demonstrated that the amount of PPF at an excitatory synapse is inversely correlated to the initial release probability of neurotransmitters from the presynaptic terminal [119-121]. When the release probability at the presynaptic terminal increases, the likelihood of observing PPD or attenuated PPF also increases [120, 121]. Conversely, a decreased release probability at the presynaptic terminal results in an increased magnitude of PPF and a decreased probability of observing PPD [121]. The PPD and reduced PPF observed in the Dnmt3a OE animals likely reflect an initial elevated neurotransmitter release probability at CA3-CA1 synapses. In contrast, Dnmt3a KD in WT and 5xFAD mice showed slightly increased CA3-CA1 PPF, suggesting a decreased initial release probability. The cellular and molecular mechanisms underlying neurotransmitter release probability, and its functional implications are complex. Both increased and decreased release probability have been associated with glutamate excitotoxicity [122]. Increased release probability represents a state of hyperexcitability, whereas decreased release probability indicates that synaptic transmission can be easily facilitated by small stimulations. It is possible that the memory impairments observed in mice with Dnmt3a OE/KD and heightened anxiety observed in mice with Dnmt3a OE were mediated by glutamate excitotoxicity at the CA3-CA1 synapse. Further molecular experiments are needed to test this hypothesis.

In summary, our findings demonstrate that DNMT3a mediates MET's therapeutic effects in AD by regulating multiple pathogenic pathways. While physiological DNMT3a levels are essential for cognitive function, both deficiency and excess prove detrimental, highlighting the importance of epigenetic homeostasis. These results support methyl supplementation as a potential strategy for AD patients with DNA hypomethylation, while cautioning that precise DNMT3a modulation will be critical for therapeutic success.

While our findings provide important insights into DNA methylation dysregulation in AD pathogenesis, several limitations should be acknowledged. The most significant limitation is the absence of direct validation in human post-mortem brain tissue. While our 5xFAD mouse model recapitulates key features of human AD epigenetics, confirmation in human specimens would provide crucial validation for translational applications. Secondly, while our findings clearly implicate DNMT3a in regulating hippocampal synaptic plasticity and 5-HT signaling, the precise molecular mechanisms remain to be determined. As DNMT3a directly mediates DNA methylation—a key epigenetic regulator of gene expression—we hypothesize that its effects occur through methylation-dependent modulation of genes involved in these pathways. To establish this mechanistic link, future experiments can combine whole-genome bisulfite sequencing and RNA-sequencing in the hippocampus following DNMT3a manipulation, with targeted analysis of genes involved in synaptic plasticity and 5-HT signalling pathways. Functional validation could employ DNA methylation-editing tools (e.g., dCas9-DNMT3a [123] and dCas9-TET1 [124]) to directly link DNA methylation changes in synaptic plasticity and 5-HT signalling pathways to phenotypic outcomes. Thirdly, while our study establishes DNMT3a as a key mediator of MET’s pro-cognitive effects, other pathways likely contribute. Future studies can investigate the involvement of TET1 and GADD45B, as we observed that their mRNA levels were decreased in 5xFAD mice and rescued by MET, suggesting their roles as co-mediators of MET. Additionally, MET supplementation could influence cognition through SAM-dependent methylation of other molecular targets, such as histones and phospholipids [125]. Future studies can investigate how MET influence histone and phospholipid methylation in the context of AD.

## Supplementary Materials

The Supplementary data can be found online at: www.aginganddisease.org/EN/10.14336/AD.2025.0283.



## Data Availability

All data are available from the corresponding author upon request.
