# Supplementary material for: Homeostatic DNMT3a Activity Is Required to Restore Cognition and Hippocampal DNA Methylation in the 5xFAD Model of Alzheimer’s Disease
Source: Aging Dis. 2026 Jun 20;17(5):2710–39. doi: 10.14336/AD.2025.0283 (PMC13437123; doi:10.14336/AD.2025.0283)
Supplement: Supplementary file 1 [file AD-17-5-2710-s.pdf]

SUPPLEMENTARY DATA

Homeostatic DNMT3a Activity Is Required to Restore Cognition and Hippocampal DNA Methylation in the 5xFAD Model of Alzheimer's Disease

Yanzhi Liu, Chi Him Poon, Long Sum Rachel Tse, Jaydeep Roy, Charlotte Theodora So, Benjamin Chun-Kit Tong, Kwok-Ming Yao, George Lim Tipoe, Harry Steinbusch, Luca Aquili, Allan Kalueff, King-Ho Cheung, Man-Lung Fung, Lee Wei Lim

© 2025. Liu Y et al. Published online at http://www.aginganddisease.org/EN/10.14336/AD.2025.0283

SUPPLEMENTARY DATA

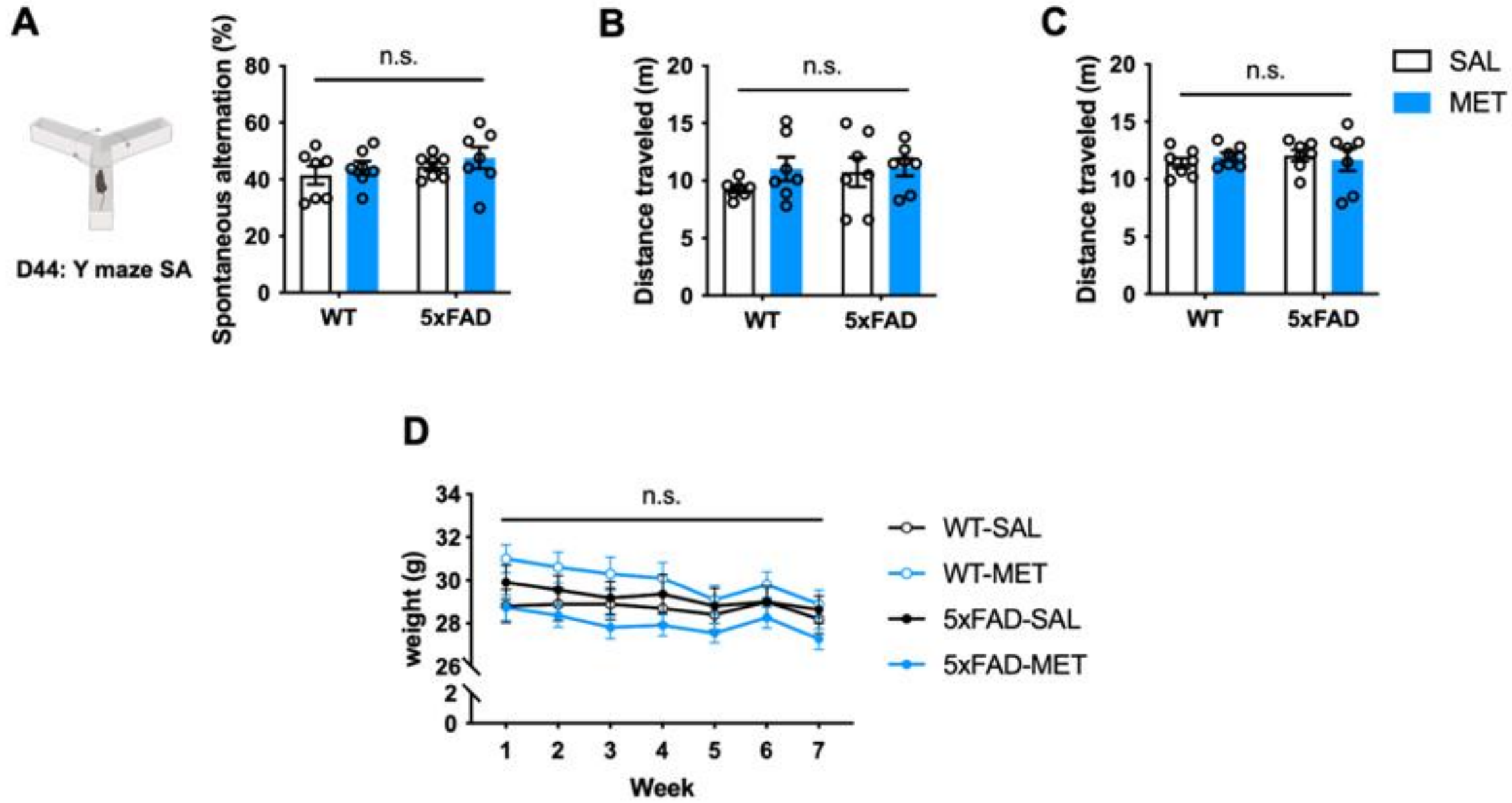

Supplementary Figure 1. Protracted MET treatment does not alter locomotor ability or weight in WT and 5xFAD mice. A Percentage of spontaneous alternations in the Y maze spontaneous alternation test (N = 7 per group) (two-way ANOVA: n.s.)**. B-C** Total distance traveled by the animals in the forced alternation Y maze (**B**) and the probe test of MWM (**C**) (N = 7 per group) (two-way ANOVA: n.s.). **D** Effect of protracted MET administration on animals' weight. Three-way repeated measures ANOVA revealed no significant differences in animals' weight throughout the SAL/MET treatment period. Data are presented as mean ± s.e.m. n.s., not significant.

© 2025. Liu Y et al. Published online at http://www.aginganddisease.org/EN/10.14336/AD.2025.0283

SUPPLEMENTARY DATA

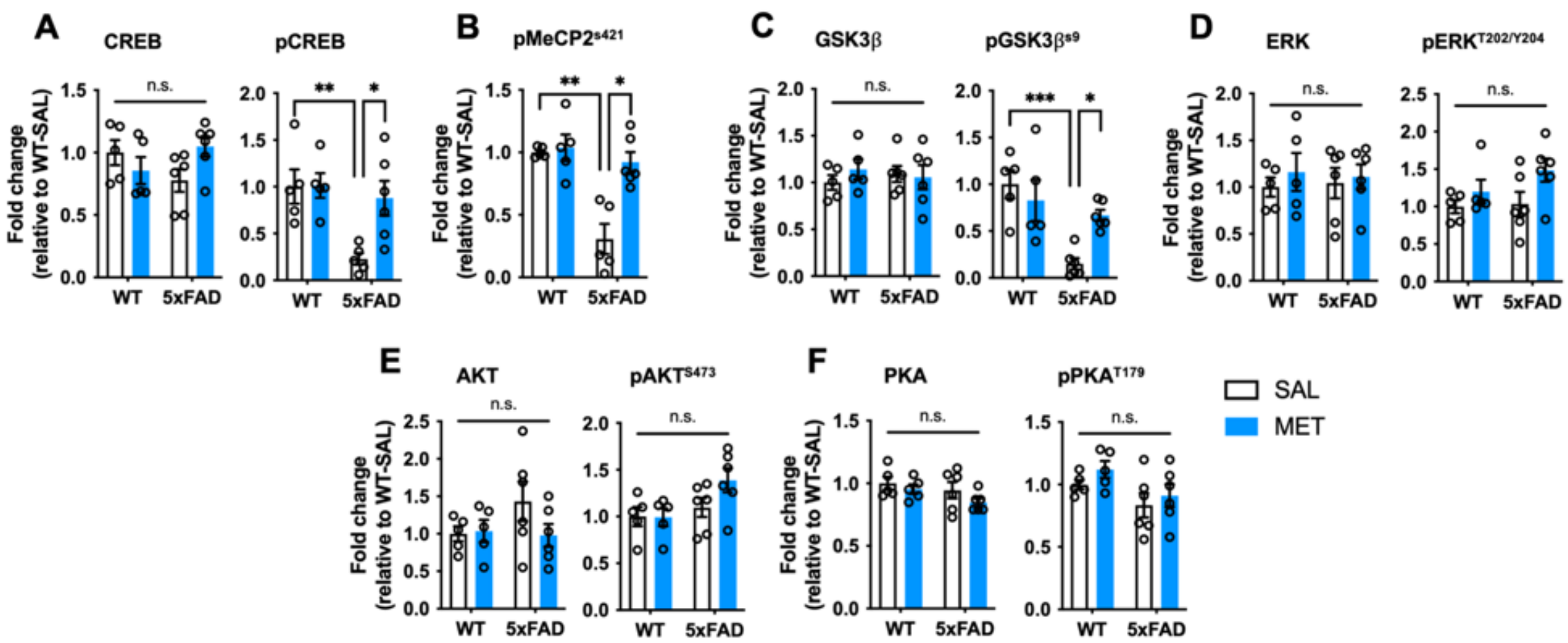

Supplementary Figure 2. Additional densitometric analysis of Western blot data shown in Figure 3. A-E Densitometric quantification of CREB, pCREB, $pMeCP2^{S421}$, GSK3β, $pGSK3β^{s9}$, ERK, $pERK^{T202/Y204}$, AKT, $pAKT^{S473}$, PKA, $pPKA^{T179}$ in the dorsal hippocampus (N = 5 per group) (two-way ANOVA with Šídák's post-hoc tests. CREB, GSK3β, ERK, AKT, $pAKT^{S473}$, PKA, $pPKA^{T179}$: n.s.; pCREB: WT-SAL vs. 5xFAD-SAL: $p = 0.018$; 5xFAD-SAL vs. 5xFAD-MET: $p = 0.044$; $pMeCP2^{S421}$: WT-SAL vs. 5xFAD-SAL: $p < 0.001$; 5xFAD-SAL vs. 5xFAD-MET: $p < 0.001$; $pGSK3β^{s9}$: WT-SAL vs. 5xFAD-SAL: $p = 0.001$; 5xFAD-SAL vs. 5xFAD-MET: $p = 0.047$; $pERK^{T202/Y204}$: 5xFAD-SAL vs. 5xFAD-MET: $p = 0.037$). Data are presented as mean ± s.e.m. *p < 0.05, ** p < 0.01, ***p < 0.001. n.s., not significant.

© 2025. Liu Y et al. Published online at http://www.aginganddisease.org/EN/10.14336/AD.2025.0283

SUPPLEMENTARY DATA

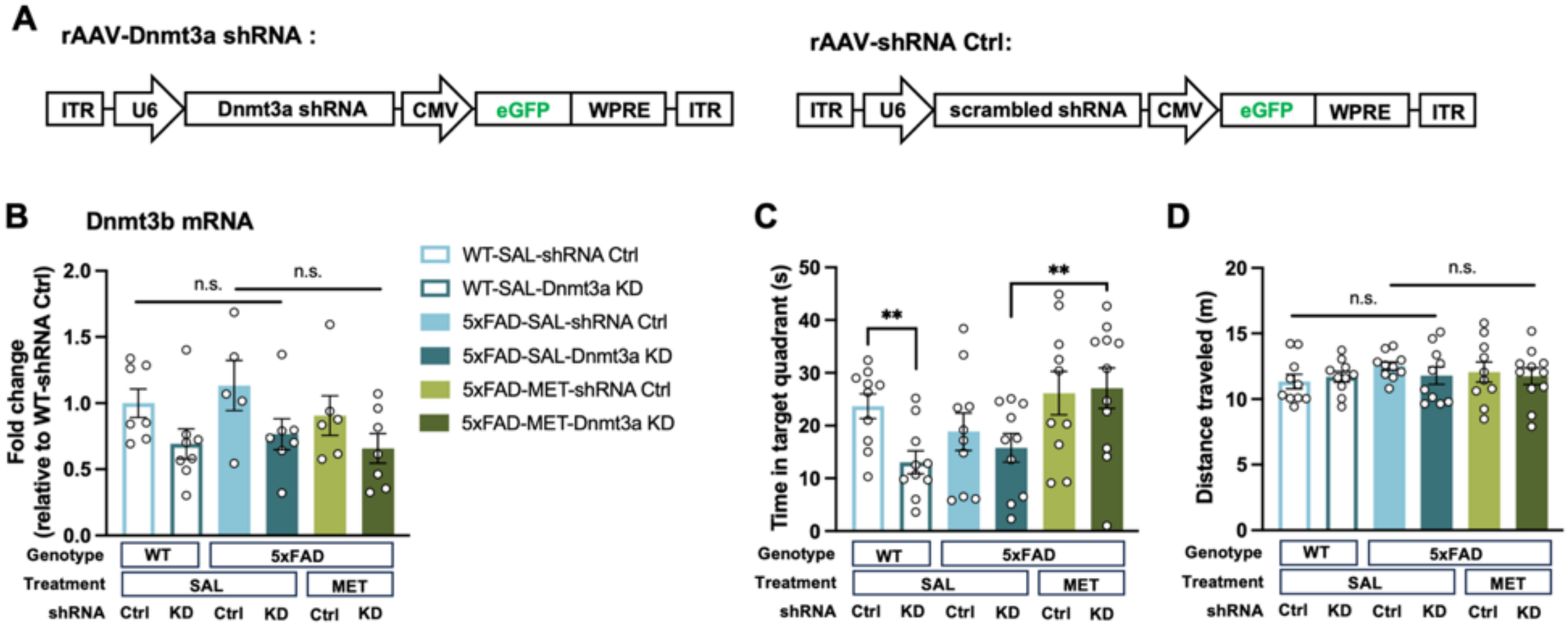

Supplementary Figure 3. Effects of Dnmt3a knockdown on Dnmt3b mRNA level and performance in Morris water maze probe test. A Schematics showing the rAAVs with Dnmt3a-targeting shRNA and control shRNA used in the current experiments. **B** RT-qPCR analysis of Dnmt3b in WT or 5xFAD mice receiving either SAL or MET treatment and either control shRNA or Dnmt3a knockdown shRNA (N = 5-8 per group) (two-way ANOVA with Šídák's post-hoc tests: n.s.)**. C-D** Time spent in the target quadrant (**C**) and total distance traveled (**D**) by animals in the probe test of MWM (N = 10 per group) (two-way ANOVA with Šídák's post-hoc: **C**: WT-SAL-shRNA Ctrl vs. WT-SAL-Dnmt3a KD: p = 0.009; 5xFAD-SAL-Dnmt3a KD vs. 5xFAD-MET-Dnmt3a KD: p = 0.007; **D**: n.s.). Data are presented as mean ± s.e.m. *p < 0.05, ** p < 0.01, ***p < 0.001. n.s., not significant.

© 2025. Liu Y et al. Published online at http://www.aginganddisease.org/EN/10.14336/AD.2025.0283

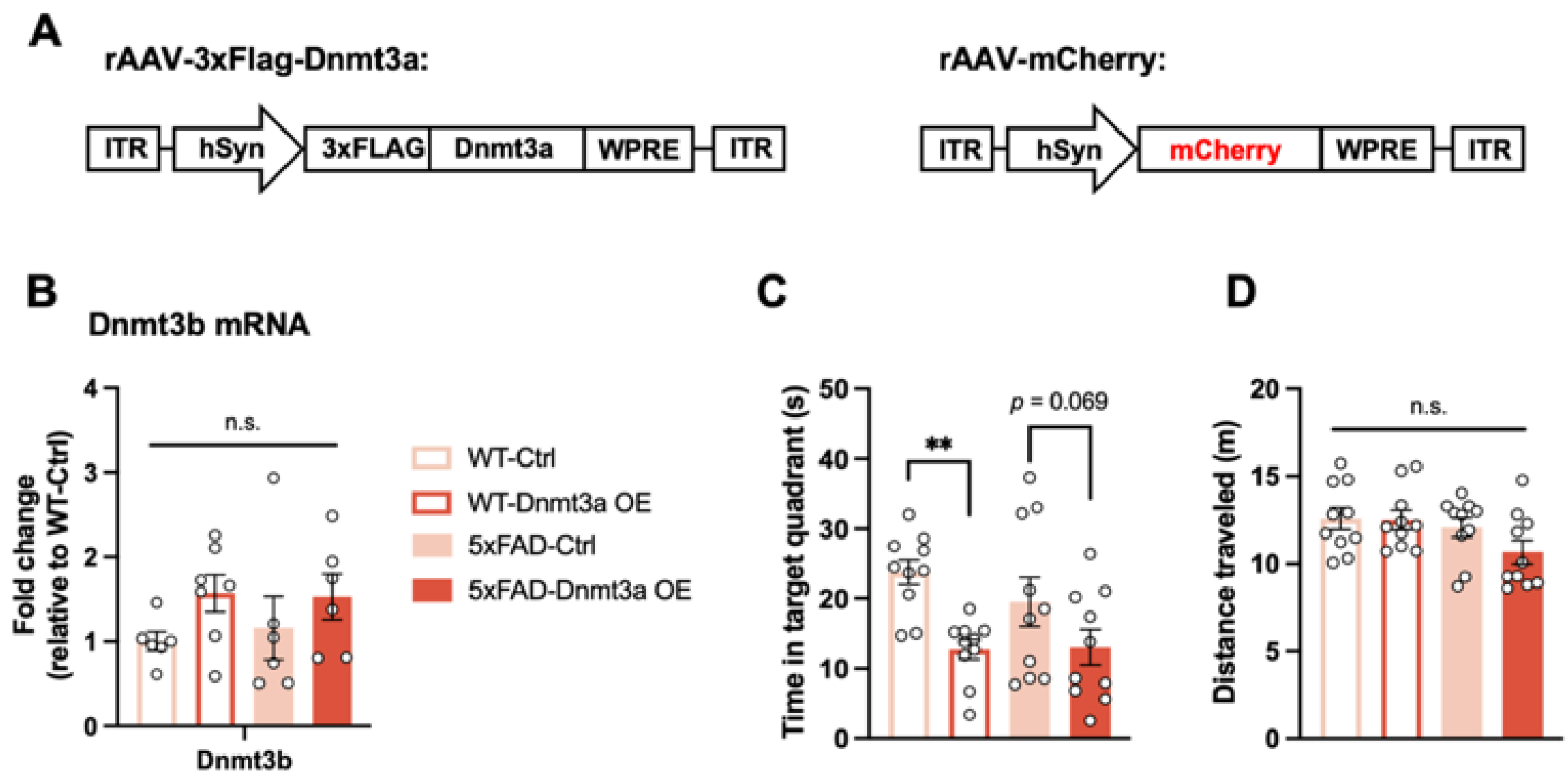

Supplementary Figure 4. Effects of Dnmt3a overexpression on Dnmt3b mRNA level and performance in Morris water maze probe test. **A** Schematics showing the rAAVs overexpressing Dnmt3a and control rAAVs used in the current experiments. **B** RT-qPCR analysis of Dnmt3b in WT or 5xFAD mice receiving either control rAAVs or Dnmt3a overexpression rAAVs (N = 6-7 per group) (two-way ANOVA with Šídák's post-hoc tests: n.s.). **C-D** Time spent in the target quadrant (**C**) and total distance traveled (**D**) by animals in the probe test of MWM (N = 10 per group) (two-way ANOVA with Šídák's post-hoc: **C**: WT-Ctrl vs. WT-Dnmt3a OE: $p = 0.003$; 5xFAD-Ctrl vs. 5xFAD-Dnmt3a OE: $p = 0.069$; **D**: n.s.). Data are presented as mean ± s.e.m. ** $p < 0.01$. n.s., not significant.

© 2025. Liu Y et al. Published online at http://www.aginganddisease.org/EN/10.14336/AD.2025.0283

SUPPLEMENTARY DATA

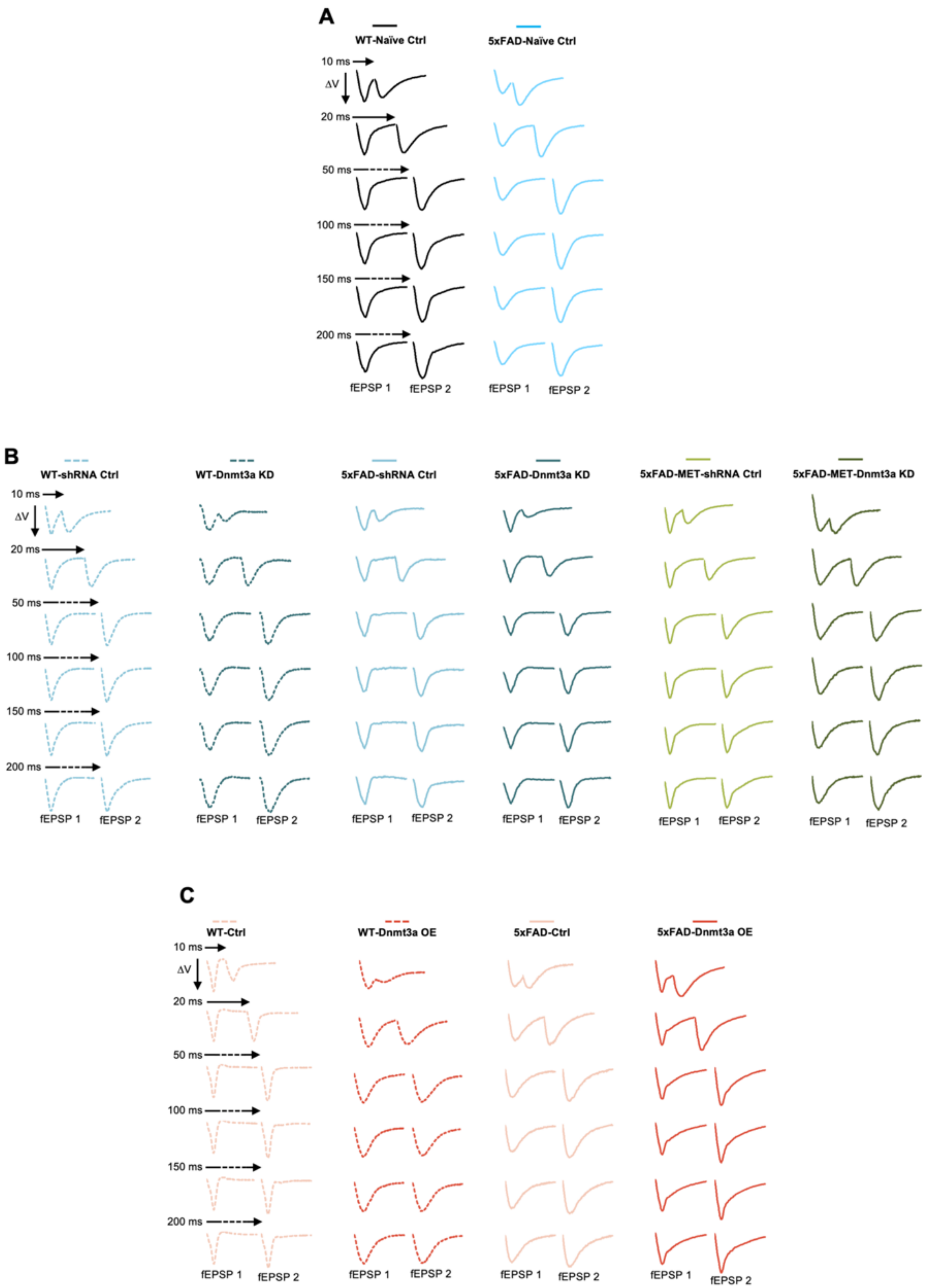

Supplementary Figure S5 Representative fEPSP traces in paired-pulse response experiments

© 2025. Liu Y et al. Published online at http://www.aginganddisease.org/EN/10.14336/AD.2025.0283

SUPPLEMENTARY DATA

A-C Representative pairs of fEPSP traces at different ISIs in naïve control groups (**A**), Dnmt3a KD groups (**B**) and Dnmt3a OE groups (**C**). Stimulus artifacts are not shown.

Supplementary Table S1 RT-qPCR primers used in the study.

Target gene	Sequence (5'-3')	Reference
Dnmt1	Forward: CCT AGT TCC GTG GCT ACG AGG AGA A<br>Reverse: TCT CTC TCC TCT GCA GCC GAC TCA	[16]
Dnmt3a	Forward: GCC GAA TTG TGT CTT GGT GGA TGA CA<br>Reverse: CCT GGT GGA ATG CAC TGC AGA AGG A	[1]
Dnmt3b	Forward: TTC AGT GAC CAG TCC TCA GAC ACG AA<br>Reverse: TCA GAA GGC TGG AGA CCT CCC TCT T	[1]
Tet1	Forward: GAG CCT GTT CCT CGA TGT GG<br>Reverse: CAA ACC CAC CTG AGG CTG TT	[5]
Tet2	Forward: TGT TGT TGT CAG GGT GAG AAT C<br>Reverse: TCA ACT TCA GGT TCC CAG TG	[5]
Tet3	Forward: CCG GAT TGA GAA GGT CAT CTA C<br>Reverse: AAG ATA ACA ATC ACG GCG TTC T	[5]
Gadd45b	Forward: TGC ATC ATG ACC CTG GAA GAG<br>Reverse: ACC GCC TGC ATC TTC TGA AC	[8]
Arc	Forward: ACG ATC TGG CTT CCT CAT TCT GCT<br>Reverse: AGG TTC CCT CAG CAT CTC TGC TTT	[3]
Reln	Forward: AGT ACT CAG ATG TGC AGT GGG CAA<br>Reverse: AGC GCT CCT TCA GGA AAG TCT TCA	[5]
CaN	Forward: AAG ATG GGA AAT GAG GCG AG<br>Reverse: CTC AGG CAG AGA CAT GAA CTC	[5]
Htr1a	Forward: GAC AGG CGG CAA CGA TAC T<br>Reverse: CCA AGG AGC CGA TGA GAT AGT T	[14]
Htr1b	Forward: GCT TTG TGA ACA CCG ACC AC<br>Reverse: GAG CCC GGG AGT TAA TGG AG	[11]
Htr1d	Forward: TGT CTC CTC CAA ACC AGT CC<br>Reverse: ACG AGC GAG ATT CTG AGA GC	[12]
Htr1f	Forward: TTT CTA CAT CCC GCT TGT AT<br>Reverse: TCG GAC AAG GAT TTT TCT AA	[15]
Htr2a	Forward: TGT GAT GCT TTT AAC ATT GC<br>Reverse: CCA ACT TAC TCC CAT GCT AC	[15]
Htr2b	Forward: GGT CAC TGG CTG CTT TCT TC<br>Reverse: TAG GCG TTG AGG TGG CTT AT	[12]
Htr2c	Forward: GTT CAA TTC GCG GAC TAA GG<br>Reverse: TCA CGA ACA CTT TGC TTT CG	[10]
Htr3a	Forward: AAC AGC TAT GCA GAA ATG AAG TT<br>Reverse: GGC TGA CTG CGT AGA ATA AAG G	[7]
Htr4	Forward: AAT GTG AGT TCC AAC GAG GG<br>Reverse: GTG AAA GAT CGA TGC TGT GG	[4]
Htr5a	Forward: AAG ACC AAC AGC GTC TCC C<br>Reverse: TCC ACG TAT CCC CTT CTG TC	[6]
Htr5b	Forward: GAG TCT GAG ATG GTG TTC A	[15]

© 2025. Liu Y et al. Published online at http://www.aginganddisease.org/EN/10.14336/AD.2025.0283

	Reverse: AAT ATC CAA GCC ACA GGA AT	
Htr6	Forward: GCA TAG CTC AGG CCG TAT GT<br>Reverse: TCC CGC ATG AAG AGG GGA TA	[13]
Htr7	Forward: TCT TCT GCA ACG TCT TCA TC<br>Reverse: ATT TCC CAT TCT GCC TCA C	[2]
GAPDH	Forward: GTC GGT GTG AAC GGA TTT G<br>Reverse: AAT TTG CCG TGA GTG GAG TC	[9]

Supplementary Table S2 Methylation specific qPCR primers used in the study

Target gene	Sequence (5' – 3')
CaN methylated	Forward: GAT AGT TGC GGT AGC GAT AG<br>Reverse: AAT ATA TAA ATA ACC CGC AAT AAT AAA TTC
CaN unmethylated	Forward: TTG GAT AGT TGT GGT AGT GA<br>Reverse: AAT ATA TAA ATA ACC CAC AAT AAT AAA TTC
Reln methylated	Forward: GGT TTG ATT GGA AAG GGT TC<br>Reverse: AAA ACC GAA AAC GAC GAA AA
Reln unmethylated	Forward: GTT TGA TTG GAA AGG GTT TG<br>Reverse: AAA ACC AAA AAC AAC AAA AA
Arc methylated	Forward: GGA GTT AGG GTT TTA CGA GTT<br>Reverse: ACT ACA ACG ACA ATA ACA AAT ATA CGA TAC
Arc unmethylated	Forward: GGA GTT AGG GTT TTA TGA GT<br>Reverse: ACT ACA ACA ACA ATA ACA AAT ATA CAA TAC
Htr1a methylated	Forward: AGT TAT TCG GGA GAG TTC GGT TTT TAC<br>Reverse: ACT TAA CTC CCG CGA ATC CTA AAC G
Htr1a unmethylated	Forward: ATT AGT TAT TTG GGA GAG TTT GGT TTT TAT<br>Reverse: CTC TTA CTT AAC TCC CAC AAA TCC TAA ACA
Htr1b methylated	Forward: GTG GGA ATT TTA GTT TTT AAT ACG G<br>Reverse: ACA AAT TCC CTA ACC TTA ATT TCG
Htr1b unmethylated	Forward: AGT GGG AAT TTT AGT TTT TAA TAT GG<br>Reverse: ACA AAT TCC CTA ACC TTA ATT TCA C
Htr5b methylated	Forward: TAA TAA GTT TAT TTG AGG GAT TTG C<br>Reverse: ATA ACT ATA ACG CAA TAC TAA CGA T
Htr5b unmethylated	Forward: ATA AGT TTA TTT GAG GGA TTT GTG G<br>Reverse: AAA AAC TAT AAC TAT AAC ACA ATA CTA ACA
GAPDH unmethylated	Forward: GTA TTG GTT TAG TAA AGA GGT G<br>Reverse: AAA ATT CTT CAC TCC AAA AA

© 2025. Liu Y et al. Published online at http://www.aginganddisease.org/EN/10.14336/AD.2025.0283

SUPPLEMENTARY DATA

Supplementary Table S3 Statistical tests and adjustments

Figure	Test	Post-hoc comparisons	Adjustment method	Number of comparisons in each adjustment
**1b-d**	Two-way ANOVA (2 x 2)	Pair-wise comparison	Fisher's LSD	6
**1e**	Three-way ANOVA (2 x 2 x 4 time points)	Pair-wise comparison at each time point	Fisher's LSD	6
**1h**	Man-Whitney U tests	N/A	N/A	N/A
**1j**	Two-way ANOVA (2 x 2)	Pair-wise comparison	Šídák's	6
**2a-k**	Two-way ANOVA (2 x 2)	Pair-wise comparison	Šídák's	6
**2l-n**	Kruskal-Wallis H test	Pair-wise comparison	Dunn's	3
**3b-n**	Two-way ANOVA (2 x 2)	Pair-wise comparison	Šídák's	6
**4a-c**	Two-way ANOVA (2 x 2)	Pair-wise comparison	Fisher's LSD	6
**4g-i**	Two-way ANOVA (2 x 2)	Pair-wise comparison	Šídák's	6
**4j-l**	Kruskal-Wallis H test	Pair-wise comparison	Dunn's	3
**5 c-d**	Two-way ANOVA (2 x 2)	Pair-wise comparison	Fisher's LSD	6
**5e-f**	Three-way ANOVA (2 x 2 x 4 time points)	Pair-wise comparison at each time point	Fisher's LSD	6
**5g-o**	Two-way ANOVA (2 x 2)	Pair-wise comparison	Šídák's	6
**6a-h**	Two-way ANOVA (2 x 2)	Pair-wise comparison	Šídák's	6
**7c-d**	Two-way ANOVA (2 x 2)	Pair-wise comparison	Šídák's	6
**7e**	Three-way ANOVA (2 x 2 x 4 time points)	Pair-wise comparison at each time point	N/A	N/A
**7f**	Two-way ANOVA (2 x 2)	Pair-wise comparison	Šídák's	6
**7h-j**	Man-Whitney U tests	N/A	N/A	N/A
**7l-n**	Two-way ANOVA (2 x 2)	Pair-wise comparison	Šídák's	6
**8a-h**	Two-way ANOVA (2 x 2)	Pair-wise comparison	Šídák's	6
**9b-e**	Two-way ANOVA (2 x 2)	Pair-wise comparison	Šídák's	6
	Paired samples t-tests	N/A	N/A	N/A
**9 f-k**	Two-way ANOVA (2 x 2)	Pair-wise comparison	Šídák's	6

References

1 Anier K, Malinovskaja K, Aonurm-Helm A, Zharkovsky A, Kalda A (2010) DNA methylation regulates cocaine-induced behavioral sensitization in mice. Neuropsychopharmacology 35: 2450-2461 Doi 10.1038/npp.2010.128

2 Arbabi Jahan A, Rad A, Ghanbarabadi M, Amin B, Mohammad-Zadeh M (2018) The role of serotonin and its receptors on the anticonvulsant effect of curcumin in pentylenetetrazol-induced seizures. Life Sciences 211: 252-260 Doi https://doi.org/10.1016/j.lfs.2018.09.007

3 Greer CB, Wright J, Weiss JD, Lazarenko RM, Moran SP, Zhu J, Chronister KS, Jin AY, Kennedy AJ, Sweatt JDet al (2021) Tet1 Isoforms Differentially Regulate Gene Expression, Synaptic Transmission, and Memory in the Mammalian Brain. J Neurosci 41: 578-593 Doi 10.1523/jneurosci.1821-20.2020

4 Ishida Y, Sugiura Y, Magome T, Kamakura T, Takimoto Y, Hanada Y, Kitayama K, Nakamura Y, Shimada S, Ohta Net al (2019) Expression Analysis of Serotonin Receptors, Serotonin Transporter and l-Amino Acid Decarboxylase in the Mouse Sphenopalatine Ganglion by RT-PCR, Northern Blot Analysis and In Situ Hybridization. Neuroscience 411: 23-36 Doi 10.1016/j.neuroscience.2019.05.028

5 Kaas GA, Zhong C, Eason DE, Ross DL, Vachhani RV, Ming GL, King JR, Song H, Sweatt JD (2013) TET1 controls CNS 5-methylcytosine hydroxylation, active DNA demethylation, gene transcription, and memory formation. Neuron 79: 1086-1093 Doi 10.1016/j.neuron.2013.08.032

© 2025. Liu Y et al. Published online at http://www.aginganddisease.org/EN/10.14336/AD.2025.0283

6 Konthapakdee N, Grundy L, O'Donnell T, Garcia-Caraballo S, Brierley SM, Grundy D, Daly DM (2019) Serotonin exerts a direct modulatory role on bladder afferent firing in mice. J Physiol 597: 5247-5264 Doi 10.1113/jp278751
7 Larson ED, Vandenbeuch A, Voigt A, Meyerhof W, Kinnamon SC, Finger TE (2015) The Role of 5-HT3 Receptors in Signaling from Taste Buds to Nerves. J Neurosci 35: 15984-15995 Doi 10.1523/jneurosci.1868-15.2015
8 Li J, Chen B, Zhong L, Gao F, Zhu H, Wang F (2018) AMP-activated protein kinase agonist N(6)-(3-hydroxyphenyl)adenosine protects against fulminant hepatitis by suppressing inflammation and apoptosis. Cell Death Dis 9: 37 Doi 10.1038/s41419-017-0118-0
9 Lim LW, Shrestha S, Or YZ, Tan SZK, Chung HH, Sun Y, Lim CL, Khairuddin S, Lufkin T, Lin VCL (2016) Tetratricopeptide repeat domain 9A modulates anxiety-like behavior in female mice. Scientific Reports 6: 37568 Doi 10.1038/srep37568
10 Mikulski Z, Zaslona Z, Cakarova L, Hartmann P, Wilhelm J, Tecott LH, Lohmeyer J, Kummer W (2010) Serotonin activates murine alveolar macrophages through 5-HT2C receptors. Am J Physiol Lung Cell Mol Physiol 299: L272-280 Doi 10.1152/ajplung.00032.2010
11 Shin HC, Lee JH, Kim KJ, Shin HJ, Choi JJ, Lee CY, Namgung U, Jung IC (2017) Modulation of hippocampal neuronal activity by So-ochim-tang-gamibang in mice subjected to chronic restraint stress. BMC Complement Altern Med 17: 456 Doi 10.1186/s12906-017-1963-1
12 Shuai J, Gao Y, Chen L, Wang Z (2021) Role of serotonin in regulation of pancreatic and mesenteric arterial function in diabetic mice. Eur J Pharmacol 901: 174070 Doi 10.1016/j.ejphar.2021.174070
13 Sun Y, Lu W, Du K, Wang JH (2019) microRNA and mRNA profiles in the amygdala are relevant to fear memory induced by physical or psychological stress. J Neurophysiol 122: 1002-1022 Doi 10.1152/jn.00215.2019
14 Svirin E, Veniaminova E, Costa-Nunes JP, Gorlova A, Umriukhin A, Kalueff AV, Proshin A, Anthony DC, Nedorubov A, Tse ACKet al (2022) Predation Stress Causes Excessive Aggression in Female Mice with Partial Genetic Inactivation of Tryptophan Hydroxylase-2: Evidence for Altered Myelination-Related Processes. Cells 11: Doi 10.3390/cells11061036
15 Vogelgesang S, Niebert S, Renner U, Möbius W, Hülsmann S, Manzke T, Niebert M (2017) Analysis of the Serotonergic System in a Mouse Model of Rett Syndrome Reveals Unusual Upregulation of Serotonin Receptor 5b. Front Mol Neurosci 10: 61 Doi 10.3389/fnmol.2017.00061
16 Yin S, Zhang Q, Yang J, Lin W, Li Y, Chen F, Cao W (2017) TGFβ-incurred epigenetic aberrations of miRNA and DNA methyltransferase suppress Klotho and potentiate renal fibrosis. Biochim Biophys Acta Mol Cell Res 1864: 1207-1216 Doi 10.1016/j.bbamcr.2017.03.002

© 2025. Liu Y et al. Published online at http://www.aginganddisease.org/EN/10.14336/AD.2025.0283
